# Roles of distal aspartate and arginine of B-class dye-decolorizing peroxidase in heterolytic hydrogen peroxide cleavage

**DOI:** 10.1074/jbc.RA118.004773

**Published:** 2018-08-02

**Authors:** Vera Pfanzagl, Kevin Nys, Marzia Bellei, Hanna Michlits, Georg Mlynek, Gianantonio Battistuzzi, Kristina Djinovic-Carugo, Sabine Van Doorslaer, Paul G. Furtmüller, Stefan Hofbauer, Christian Obinger

**Affiliations:** From the ‡ Department of Chemistry, Division of Biochemistry, BOKU–University of Natural Resources and Life Sciences, 1190 Vienna, Austria,; the § Department of Physics, University of Antwerp, 2610 Wilrijk, Belgium,; the Departments of ¶ Life Sciences and; ** Chemistry and Geology, University of Modena and Reggio Emilia, via Campi 103, 41125 Modena, Italy, and; the ‖ Department for Structural and Computational Biology, Max F. Perutz Laboratories, University of Vienna, 1030 Vienna, Austria

**Keywords:** heme, enzyme kinetics, Klebsiella pneumonia, pre-steady-state kinetics, site-directed mutagenesis, X-ray crystallography, electron paramagnetic resonance (EPR), Compound I, Compound II, heme peroxidase, oxoiron

## Abstract

Dye-decolorizing peroxidases (DyPs) represent the most recently classified hydrogen peroxide–dependent heme peroxidase family. Although widely distributed with more than 5000 annotated genes and hailed for their biotechnological potential, detailed biochemical characterization of their reaction mechanism remains limited. Here, we present the high-resolution crystal structures of WT B-class DyP from the pathogenic bacterium *Klebsiella pneumoniae* (*Kp*DyP) (1.6 Å) and the variants D143A (1.3 Å), R232A (1.9 Å), and D143A/R232A (1.1 Å). We demonstrate the impact of elimination of the DyP-typical, distal residues Asp-143 and Arg-232 on (i) the spectral and redox properties, (ii) the kinetics of heterolytic cleavage of hydrogen peroxide, (iii) the formation of the low-spin cyanide complex, and (iv) the stability and reactivity of an oxoiron(IV)porphyrin π-cation radical (Compound I). Structural and functional studies reveal that the distal aspartate is responsible for deprotonation of H_2_O_2_ and for the poor oxidation capacity of Compound I. Elimination of the distal arginine promotes a collapse of the distal heme cavity, including blocking of one access channel and a conformational change of the catalytic aspartate. We also provide evidence of formation of an oxoiron(IV)-type Compound II in *Kp*DyP with absorbance maxima at 418, 527, and 553 nm. In summary, a reaction mechanism of the peroxidase cycle of B-class DyPs is proposed. Our observations challenge the idea that peroxidase activity toward conventional aromatic substrates is related to the physiological roles of B-class DyPs.

## Introduction

Dye-decolorizing peroxidases (DyPs) ^2^ are heme *b*–containing oxidoreductases that catalyze the hydrogen peroxide–mediated oxidation of various substrates. Their discovery and characterization (1, 2) led to their classification as a novel heme peroxidase family with predominantly bacterial origin (3). These metalloenzymes have a distinct evolutionary origin, tertiary structure, and active-site architecture that significantly differ from members of other heme peroxidase superfamilies (4). Phylogenetic analysis shows three distinct phylogenetic classes, called A, B, and C/D, which differ in structure, oligmeric state, and predicted localization (5). Except for class C/D, where a role in lignin degradation is under discussion, the biological substrates are unknown. The enzyme investigated in this work originates from the human pathogen *Klebsiella pneumoniae* and belongs to class B.

Similar to classes A and C/D (1, 2), all described B-class DyPs (*Rodococcus jostii* (6, 7), *Pseudomonas putida* (8), *Escherichia coli* (9), *Vibrio cholerae* (10), and *Enterobacter lignolyticus* (11)) exhibit peroxidase activities with conventional substrates (2,2-azinobis(3-ethylbenzothiazoline-6-sulfonic acid) (ABTS), guaiacol, pyrogallol, veratryl alcohol, hydroquinones, etc.), antraquinone-derived dyes (*e.g.* Reactive Blue 19), Mn(II) ions, or even lignin model compounds (6, 10–13). However, compared with other heme peroxidase families, the reported *k*_cat_/*K_m_* values are relatively small, thus raising the question of *in vivo* electron donors. Diverse enzymatic functions have been proposed for B-class DyPs, including deferrochelatase (9), porphyrin oxidase (14), or lignin peroxidase activity (12). However, to a great extent, direct biochemical and biophysical evidence of the suggested functions is missing.

In all heme peroxidase (super)families (4), the peroxidase cycle is initiated by deprotonation of hydrogen peroxide mediated by a distal base (B:) and formation of a short-lived ferric hydroperoxide (Fe(III)-O-O-H) complex, often designated as Compound 0 (Reaction 1). Heterolytic cleavage of the O–O bond mediates the two-electron oxidation of Compound 0 to Compound I, thereby forming an oxoiron(IV) species (Fe(IV)=O) in combination with either a porphyryl radical (Por^•+^) (Reaction 2) or a distinct amino acid radical (aa^•+^) (Reaction 3). Additionally, water is released.

$$\begin{array}{c} \text{B}:\;+\left(\text{Fe}\left(\text{III}\right)\text{Por}\;\ldots \;\text{aa}\right)+\text{H}-\text{O}-\text{O}-\text{H}\to \text{BH}+\left(\text{Fe}\left(\text{III}\right)-\text{O}-\text{O}-\text{H}\;\text{Por}\;\ldots \;\text{aa}\right)\\ \text{BH}\;+\left(\text{Fe}\left(\text{III}\right)-\text{O}-\text{O}-\text{H}\;\text{Por}\;\ldots \;\text{aa}\right)\to \text{B}:+\left(\text{Fe}\left(\text{IV}\right)\text{=O}\;{\text{Por}}^{\cdot +}\;\ldots \;\text{aa}\right)+{\text{H}}_{2}\text{O}\\ \text{BH}\;+\left(\text{Fe}\left(\text{III}\right)-\text{O}-\text{O}-\text{H}\;\text{Por}\;\ldots \;\text{aa}\right)\to \text{B}:+\left(\text{Fe}\left(\text{IV}\right)\text{=O}\;\text{Por}\;\ldots \;{\text{aa}}^{\cdot +}\right)+{\text{H}}_{2}\text{O}\\ \text{Reaction}\;1-3 \end{array}$$

In two heme peroxidase superfamilies (peroxidase-catalase and peroxidase-cyclooxygenase superfamilies) (4), Compound I formation is described to involve acid–base catalysis through the highly conserved His–Arg couple in the distal side, known as the Poulus–Kraut mechanism (15, 16). In all DyP classes, an Asp–Arg couple is found at the distal side (4). However, the distinct functional role of the distal catalytic amino acids in the heterolytic cleavage of H_2_O_2_ is still under dispute. In B-class DyPs, both the Arg (DyPs from *R. jostii* RHA1 (7) and *P. putida* (8)) and the Asp (Dyp from *E. lignolyticus*, *El*DyP (11)) have been proposed to act as proton acceptor and donor in Compound I formation. In A and C/D classes, the distal Asp was suggested to act as acid–base catalyst (3, 17). Likewise, the mechanism of Compound I reduction and the electronic features of Compound II of B-class DyPs remain elusive. Based on studies of solvent isotope and viscosity effects on Compound I reduction of *El*DyP, it was proposed that the reducing substrate binds to Compound I instead of free enzyme and mediates its two-electron reduction without formation of an oxoiron(IV)-type Compound II (11).

Here, we hypothesize that the distal aspartate acts as proton acceptor during heterolytic cleavage of hydrogen peroxide. This hypothesis is based on the facts that (i) B-class DyPs typically exhibit pH optima of enzymatic activity below pH 7 and deprotonation of a guanidinium group (p*K_a_* = 13.8) (18) *per se* below pH 10 is thermodynamically unfavorable even in a hydrophobic microenvironment (18) and (ii) the distal Arg in B-class DyPs forms a salt bridge with propionate p7 of the prosthetic group, which favors the protonated state of the guanidinium group to a greater extent. To confirm this hypothesis, we elucidate the crystal structures of WT *Kp*DyP, the single variants D143A and R232A as well as the double variant D143A/R232A, and perform a comprehensive biochemical and biophysical characterization of those proteins. Our study confirms the essential function of Asp-143 in hydrogen peroxide reduction and of Arg-232 in maintenance of the distal architecture. Compound I of B-class DyPs is shown to be an oxoiron(IV)porphyrin π-cation radical of very poor oxidation capacity, whereas substitution of Asp-143 significantly increases the two- and one- electron oxidation capacity of Compound I. Furthermore, we demonstrate the formation of an oxoiron(IV)-type Compound II for both WT *Kp*DyP and the variant D143A.

## Results

### Recombinant KpDyP is a stable homodimeric high-spin ferric heme protein

As a basis for elucidation of the role of Asp-143 and Arg-232 in heterolytic cleavage of H_2_O_2_, we aimed at characterization of the solution and crystal structures of WT *Kp*DyP and its variants D143A, R232A, and D143A/R232A. Consequently, these proteins were expressed heterologously in *E. coli* and purified by affinity and size-exclusion chromatography (SEC) as described under “Experimental procedures.” Typically, 60–80 mg of WT and variant heme protein per liter of culture broth were obtained. HPLC-SEC combined with multiangle light scattering confirmed the presence of pure homodimeric WT and variant proteins (2 × 35 kDa) (data not shown), which is in accordance with previously published B-class DyPs. The average Reinheitszahl (*A*_Soret_/*A*_280_) was found to be 2.1 (WT *Kp*DyP), 1.7 (D143A), and 2.7 (R232A and D143A/R232A), respectively. Upon using the pyridine-hemochromogen assay (19), the extinction coefficients at the Soret maxima were determined to be 160,000 m^−1^ cm^−1^ (WT *Kp*DyP and D143A) and 240,000 m^−1^ cm^−1^ (R232A and D143A/R232A), respectively.

**Figure 1.**
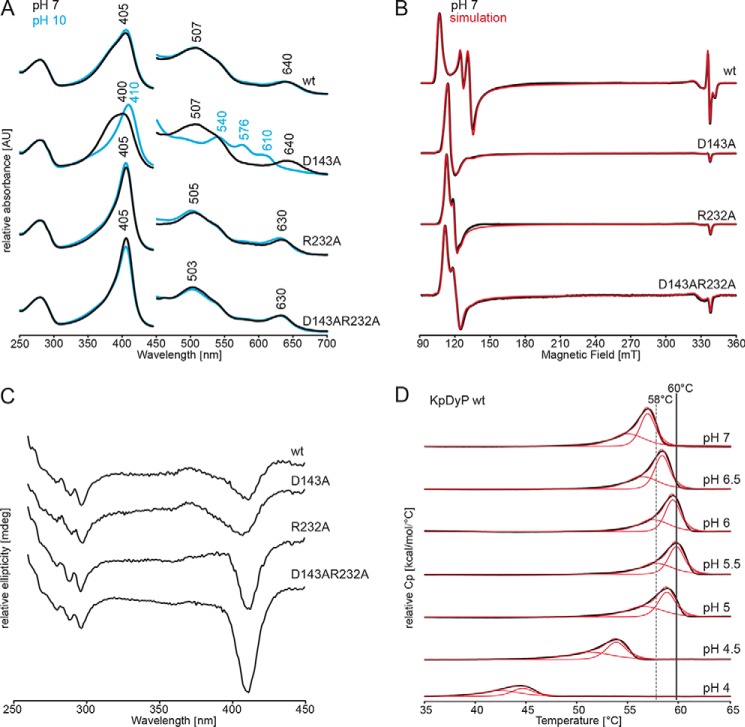
**Spectral properties and thermal stabilities of WT *Kp*DyP and the variants D143A, R232A, and D143A/R232A.** *A*, UV-visible spectra of WT *Kp*DyP, D143A, R232A, and D132A/R232A in 50 mm phosphate buffer, pH 7.0 (*black*), and in 50 mm borate buffer pH 10.0 (*blue*). *B*, low temperature X-band CW EPR spectra of WT *Kp*DyP and variants in 50 mm phosphate buffer, pH 7.0 (*black*), and their corresponding spectral simulations (*red*). *C*, CD spectra of WT *Kp*DyP and variants in 50 mm phosphate buffer, pH 7.0, in the near-UV region (260–450 nm). *D*, DSC thermograms of WT *Kp*DyP between pH 4.0 and 7.0 (*black*). The corresponding fits using a non-two-state transition model are depicted in *red*. The highest *T_m_* values (pH 5.5) are highlighted with *gray lines*.

Fig. 1 (*A* and *B*) depict the UV-visible and EPR spectral signatures of WT *Kp*DyP and the variants. At pH 7.0, all proteins are in the high-spin (HS) state. WT *Kp*DyP exhibits UV-visible bands at 405 nm (Soret band) together with a small shoulder at 385, 507, and 539 nm (Q-bands) and 640 nm (charge transfer (CT) band). Except for the broad Soret maximum at 400 nm, the spectrum of the D143A variant is identical with that of the WT protein. In the variants R232A and D143A/R232A, the CT band is blue-shifted to 630 nm, whereas the other bands are almost WT-like (R232A: 405, 505, and 539 nm; double variant: 405, 503, and 539 nm). Except for the D143A variant, no spectral changes were observed between pH 4.5 and pH 10. In the case of D143A, an alkaline transition occurred (p*K_a_* = 8.6), resulting in a species with Soret maximum at 410 nm and additional bands at 540, 576, and 610 nm, indicative of the presence of a HS hydroxo ligand (Fig. 1*A* and Fig. S1) or a mixed population of HS and LS hydroxo ligated heme iron.

The effect of the catalytic amino acids on the heme surrounding and electronic state of the heme iron of *Kp*DyP was further investigated using low-temperature continuous-wave (CW) EPR spectroscopy. The experimental spectra of ferric WT *Kp*DyP and its distal side variants at neutral pH (Fig. 1*B*) clearly display the presence of HS (*S* = 52) Fe(III) heme centers, which was not influenced by pH (data not shown). Due to the large zero-field splitting, the X-band EPR spectra can be simulated in two ways (20). On the one hand, they can be described as a *S* = 52 system with g values close to 2 and varying *E*/*D* values (with *D* and *E* being the tetragonal and rhombic zero-field splitting, respectively). On the other hand, the EPR spectra can also be simulated assuming an apparent *S* = ½ system with effective g^eff^ tensor. Both approaches were assumed here, to allow comparison with literature data (Table 1). For each *Kp*DyP variant, the presence of multiple HS species with varying rhombicity was found, as reflected by the different *E/D* values. Table 1 and Fig. 1*B* reveal that site-directed mutation of the catalytic amino acids Arg-232 and Asp-143 in *Kp*DyP strongly influences the EPR spectra, implying a marked change of the heme pocket sensed by the heme iron. Even though the heme-pocket heterogeneity remains, the dominant contribution appears to be much less rhombic.

**Table 1:** **EPR simulation parameters of spectra from WT and *Kp*DyP variants (experimental error: ±0.01 for the g values, ±0.0005 for the *E/D* ratio, and ±1% for the contribution) compared with EPR data of other heme peroxidases** HRP, horseradish peroxidase; *Mt*KatG, catalase-peroxidase from *M. tuberculosis*; *Ma*Cld, chlorite dismutase from *Magnetospirillum* sp.; *C*Cld, chlorite dismutase from the cyanobacterium *Cyanothece* sp. PCC7425; *Tc*DyP, dye-decolorizing peroxidase from *T. curvata*; *Aau*DyP, dye-decolorizing peroxidase from *A. auricula-judae*; *Nd*Cld, chlorite dismutase from *N. defluvii*; *Rj*DyP, dye-decolorizing peroxidase from *R. jostii* RHA1; TBD, to be determined; NR, not reported.

	g*_x_*^eff^	g*_y_*^eff^	g*_z_*^eff^	g*_x_*	g*_y_*	g*_z_*	*E/D*	*I*
								%
***Kp***DyP WT (this work)								
HS1	6.44	5.12	1.98	1.93	1.93	2	0.0288	77.3
HS2	6.25	5.41	1.99	1.95	1.95	2	0.0178	22
Radical				TBD	TBD	TBD		0.7

***Kp***DyP R232A (this work)								
HS1	6.06	5.69	1.99	1.96	1.96	2	0.0076	67.2
HS2	6.2	5.52	1.99	1.95	1.95	2	0.0148	23.9
HS3	6.05	5.88	2	1.99	1.99	2	0.0036	8.9

***Kp***DyP D143A (this work)								
HS1	5.99	5.85	2	1.97	1.97	2	0.0029	40.7
HS2	6.13	5.69	1.94	1.97	1.97	2	0.0097	48.2
HS3	6.43	5.15	1.97	1.94	1.94	2	0.0279	11.1

***Kp***DyP D143A/R232A (this work)								
HS1	6.13	5.61	1.99	1.96	1.96	2	0.0086	91.7
HS2	6.49	5.21	1.98	1.96	1.96	2	0.0220	8.3

**HRP (65)**								
HS	6.35	5.65	2	NR	NR	NR	NR	100

***Mt***KatG (29)								
HS1	5.86	5.86	2	NR	NR	NR	NR	NR
HS2	6.03	5.55	2	NR	NR	NR	NR	NR
HS3	6.35	5.37	2	NR	NR	NR	NR	NR
HS4	6.49	5.1	2	NR	NR	NR	NR	NR

***Ma***Cld (30)								
HS1	6.72	5.05	1.96	1.97	1.97	2	0.0380	6.5
HS2	6.29	5.45	1.99	1.97	1.97	2	0.0177	93.5

***C***Cld (32)								
HS1	5.93	5.8	2	NR	NR	NR	0.002	62
HS2	6.05	5.65	2	NR	NR	NR	0.008	28
LS				2.8	2.28	1.85		10

***Tc***DyP A class*^a^* (23)								
HS	6.2	5.5	2	NR	NR	NR	NR	100

***Aau***DyP A class pH 3*^a^* (22)								
HS1	5.9	5.9	2	NR	NR	NR	NR	NR
HS2	6.2	5.4	2	NR	NR	NR	NR	NR

***Nd***Cld (66)								
HS1	5.50	6.17	1.99	NR	NR	NR	0.014	49
HS2	5.95	5.95	1.99	NR	NR	NR	NR	19
LS1				2.98	2.25	1.88		15
LS2				3.15	2.25	1.80		16

***Rj***DyP A class*^a^* (6)								
HS	6.32	5.45	1.97	NR	NR	NR	NR	NR

***Rj***DyP B class*^a^* (6)								
HS	6.09	5.45	1.99	NR	NR	NR	NR	NR

*^a^* Readout values.

Exclusively in the case of ferric WT *Kp*DyP, an organic radical is also observed (Fig. 1*B*). The features and g-anisotropy (g values in the 2.002–2.009 range) of this EPR signal (see *top inset* of Fig. 5) suggest the presence of one or more tyrosyl radicals (21, 22). Although amino acid radicals have been observed in DyPs after activation of the enzyme with H_2_O_2_, they have, to our knowledge, never been reported for the resting state of the enzyme (22–25).

The electronic CD (ECD) spectra in the far-UV (190–250 nm; data not shown) and near-UV region (250–350 nm) of the four proteins were almost identical, suggesting that the mutations did not change the overall secondary and tertiary structure. By contrast, ECD spectra in the visible region showed differences, again reflecting altered heme cavity architectures in the four proteins. At pH 7.0, WT *Kp*DyP shows a Soret minimum at 411 nm, whereas in the variant D143A, the minimum is broadened and shifted to 407 nm. Additionally, both WT *Kp*DyP and D143A exhibit a small peak around 373 nm. In the case of the variants R232A and D143A/R232A, the ellipticity at the sharpened minimum at 411 nm is increased (Fig. 1*C*).

**Figure 2.**
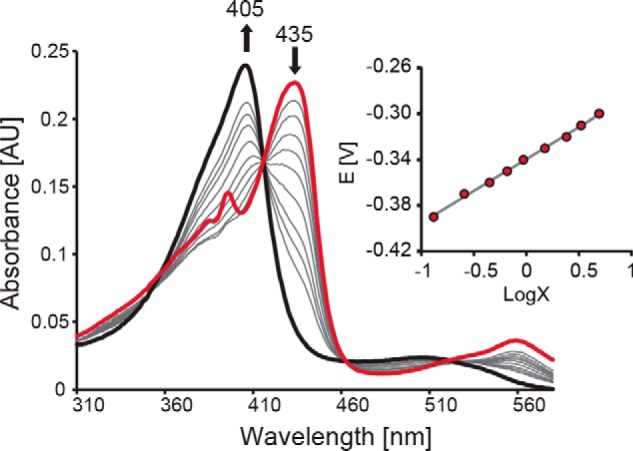
**Spectroelectrochemical titration of WT *Kp*DyP.** Shown are electronic absorption spectra of WT *Kp*DyP at different applied potentials. The *bold black spectrum* represents fully oxidized, ferric enzyme (Soret maximum at 405 nm), and the *red* spectrum represents fully reduced (Soret maximum at 435 nm) protein. The *inset* shows the corresponding Nernst plot, where *x* represents (*A*_λredMax_ − *A*_λred_)/(*A*_λoxMax_ − *A*_λ_ox).

Next, we probed the standard reduction potential (*E*°′) of the Fe(III)/Fe(II) redox couple of *Kp*DyP and its catalytic variants by spectroelectrochemical titration at pH 7.0. Upon reduction of ferric to ferrous WT *Kp*DyP, the Soret peak shifts from 405 to 435 nm. Fig. 2 shows the fully oxidized and fully reduced (*black* and *red lines*, respectively) as well as the equilibrium spectra of WT *Kp*DyP (*gray lines*) at seven different applied potentials in the optical transparent thin-layer spectroelectrochemical (OTTLE) cell (25 °C, pH 7.0). The calculated reduction potential *E*°′ for the Fe(III)/Fe(II) couple, determined from the corresponding Nernst plot (Fig. 2, *inset*), was calculated to be −0.350 ± 0.010 V. Removal of Asp-143 slightly increases the standard reduction potential (D143A: −0.330 ± 0.010 V) compared with the WT protein, whereas *E*°′ is almost unaffected by elimination of R232 (R232A: −0.347 ± 0.010 V). The *E*°′ value of D143A/R232A was calculated to be −0.299 ± 0.010 V.

Finally, we probed the thermal stability of WT *Kp*DyP between pH 3 and pH 8 by differential scanning calorimetry (DSC) because DyPs are often described for optimum peroxidase activity at acidic pH (pH <3.0). As is appropriate for a cytoplasmatic protein, WT *Kp*DyP was found to be most stable between pH 5.5 and pH 7.0 (Fig. 1*D*). Temperature-mediated unfolding shows one endotherm that can be best fitted by assumption of a non-two-state transition with two consecutive irreversible unfolding events at *T_m_* values of ∼58 and ∼60 °C at pH 5.5, which cannot be separated. All variants exhibited decreased thermal stability but followed a similar unfolding profile and pH dependence. Typically, the *T_m_* values of the mutants were lowered by 5–10 °C following the hierarchy WT > D143A > R232A > D143A/R232A. Below pH 4, WT *Kp*DyP was too unstable and aggregated upon heating.

### Distal Asp-143 is essential for pH-independent Compound I formation

Before investigating Compound I formation of *Kp*DyP, we probed the kinetics and thermodynamics of the formation of the cyanide complex of *Kp*DyP and the three variants, because cyanide binding mechanistically mirrors Compound I formation. Cyanide, which at most biological pH levels is protonated (p*K_a_* = 9.14), binds as an anion to Fe(III). Thus, HCN and H_2_O_2_ need to be deprotonated by a distal base. Thereby, deprotonation of HCN mimics the committing step of H_2_O_2_-mediated Compound I formation. Upon the addition of cyanide, high-spin WT and *Kp*DyP variants are converted to a low-spin complex with a Soret maximum at 420 nm. Spectrophotometric titration of *Kp*DyP revealed that the dissociation constant (*K_D_*) of the WT protein is very high (900 ± 40 μm). Elimination of Arg-232 increased *K_D_* (2700 ± 370 μm). In the double variant, *K_D_* was determined to be 136 ± 13 μm) (data not shown). By contrast, binding affinity of cyanide was significantly increased upon elimination of the distal aspartate (D143A: 20 ± 12 μm derived from pre-steady-state kinetics).

**Figure 3.**
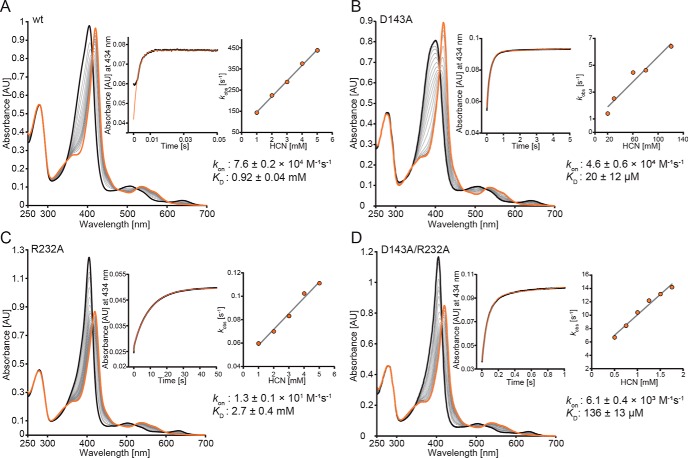
**Kinetics of cyanide binding to ferric WT *Kp*DyP and the variants D143A, R232A, and D143A/R232A at pH 7.0.** Spectral conversion of high-spin ferric protein (*black spectrum*) to the corresponding low-spin cyanide complex (*orange spectrum*) is shown. *A*, WT; *B*, D143A; *C*, R232A; *D*, D143AR232A. The *insets* show representative time traces (*black*) and fits (*orange*) at 434 nm (WT *Kp*DyP, 5 mm; D143A, 100 μm; R232A, 5 mm; D143A/R232A, 1.5 mm). Additionally, *insets* show the corresponding linear plots of *k*_obs_ *versus* cyanide concentration.

Next, we probed the kinetics of cyanide binding by stopped-flow spectroscopy. At pH 7.0, the apparent second order rate constant, *k*_on_, of the reaction between WT *Kp*DyP and cyanide was determined to be (7.6 ± 0.2) × 10^4^ m^−1^ s^−1^ (Fig. 3). Between pH 4.0 and pH 9.0, the *k*_on_ values remained almost unchanged (∼40% increase between pH 5.0 and 8.0). By contrast, cyanide binding to D143A strongly depended on pH, ranging from (5.9 ± 0.6) × 10^2^ m^−1^ s^−1^ at pH 5.0 to (2.3 ± 0.2) × 10^5^ m^−1^ s^−1^ at pH 8. At pH 7, *k*_on_ of D143A was almost WT-like ((4.6 ± 0.6) × 10^4^ m^−1^ s^−1^). Upon elimination of Arg-232, cyanide binding was drastically reduced ((13 ± 1) m^−1^ s^−1^, pH 7.0), whereas *k*_on_ for the double variant was calculated to be (6.1 ± 0.4) × 10^3^ m^−1^ s^−1^ at pH 7.0 (Fig. 3).

**Figure 4.**
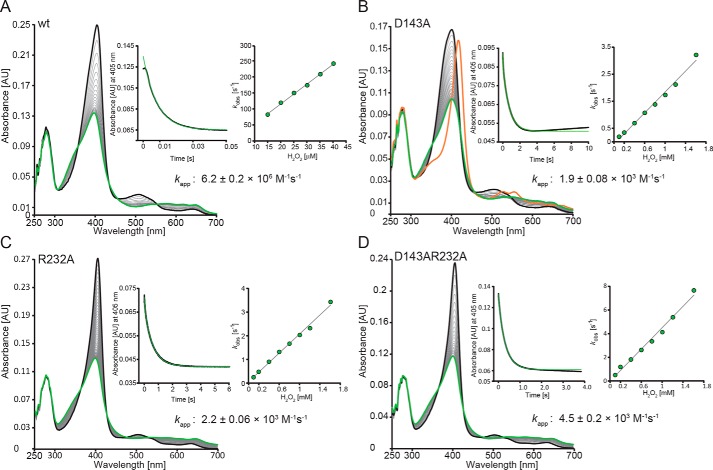
**Kinetics of Compound I formation of WT *Kp*DyP and the variants D143A, R232A, and D143A/R232A at pH 7.0.** Spectral transition upon the reaction of 2 μm WT (*A*), D143A (*B*), R232A (*C*), and D143A/R232A (*D*) with 20 μm (WT) or 800 μm (variants) hydrogen peroxide is shown. *Insets* depict typical time traces at 405 nm (*black*) and the corresponding single- or double-exponential fits (*green*). Additionally, *insets* show the corresponding linear plots of *k*_obs_ *versus* hydrogen peroxide concentration.

Next, we focused on the role of Asp-143 and Arg-232 in Compound I formation. Oxidation of WT *Kp*DyP to Compound I mediated by hydrogen peroxide was followed in the conventional stopped-flow mode (Fig. 4*A*). Compound I is characterized by a Soret maximum at 397 nm (∼50% hypochromicity compared with ferric *Kp*DyP) and formation of an additional band at 648 nm. The reaction is monophasic (*k*_app_ = (6.2 ± 0.2) ± 10^6^ m^−1^ s^−1^ at pH 7.0) with clear isosbestic points at 430 and 550 nm (Fig. 4*A*). Compound I is already formed with equimolar H_2_O_2_, and its UV-visible spectrum remains unchanged for minutes, whereas at high excess of H_2_O_2_ (>1 mm), heme is degraded within seconds, as reflected by loss of absorbance and generation of a peak at 670 nm, which might indicate biliverdin formation (26). Similar to cyanide binding, Compound I formation of WT *Kp*DyP is pH-independent between pH 4 and pH 9 (not shown).

Upon the addition of hydrogen peroxide to the variants, similar spectral transitions were observed (Fig. 4, *B–D*). However, a ∼400-fold stoichiometric excess of H_2_O_2_ is necessary to obtain full hypochromicity at the Soret band, and the apparent bimolecular rate constants are diminished by 3 orders of magnitude (*insets* to Fig. 4, *C* and *D*). Moreover, the variants R232A (*k*_app_ = (2.2 ± 0.1 × 10^3^ m^−1^ s^−1^ at pH 7.0) and D143A/R232A (*k*_app_ = (4.5 ± 0.2 × 10^3^ m^−1^ s^−1^ at pH 7.0) are significantly more prone to heme degradation compared with the WT protein. Heme degradation is reflected by the biphasic kinetics of Compound I formation, with the second slow phase representing bleaching of the prosthetic group. In the case of D143A, Compound I formation (*k*_app_ = 1.9 ± 0.1 × 10^3^ m^−1^ s^−1^ at pH 7.0) (Fig. 4*B*, *inset*) is followed by a slow H_2_O_2_-dependent conversion (*k*_app_ = 40 m^−1^ s^−1^) to a species reminiscent of classical oxoiron(IV)-type Compound II with absorbance maxima at 417, 527, and 555 nm (Fig. 4*B*, *orange spectrum*). Similar to cyanide binding, the *k*_app_ values of Compound I formation of the variant D143A increase between pH 5.0 (1.4 ± 0.1 × 10^2^ m^−1^ s^−1^) and pH 8.0 (8.6 ± 0.1 × 10^3^ m^−1^ s^−1^). By contrast, Compound I formation of R232A slightly increases with decreasing pH.

### Compound I is an oxoiron(IV) porphyrin π-cation radical

**Figure 5.**
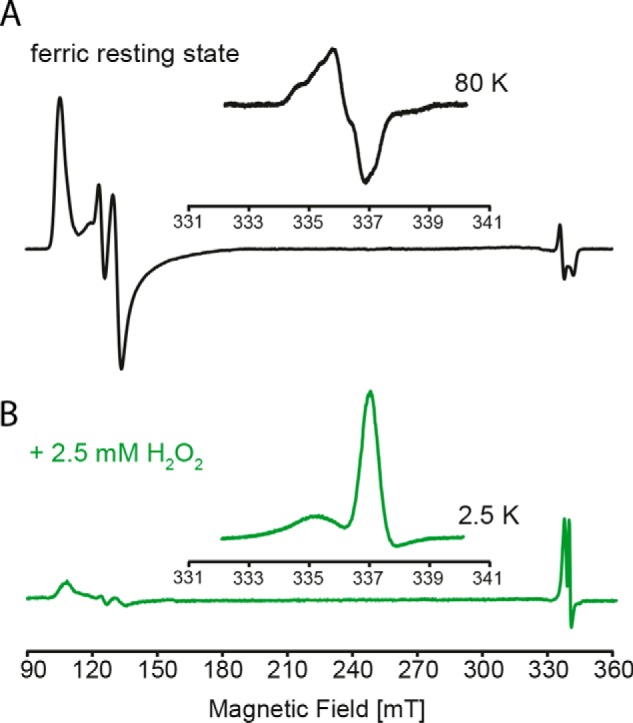
**Low temperature CW EPR of WT *Kp*DyP and Compound I.** *A*, X-band CW EPR spectrum of WT *Kp*DyP. The *inset* shows the organic radical signature of the resting state at 80 K. *B*, X-band CW EPR spectrum of *Kp*DyP Compound I formed by the addition of a 5-fold stoichiometric excess of hydrogen peroxide. The *inset* shows the porphyryl radical signature observed at 2.5 K.

Next, we probed the electronic configuration of Compound I by EPR spectroscopy at 2.5 K. Compound I was formed by the addition of a 5-fold molar excess of H_2_O_2_ to WT KpDyP (Fig. 5, *bottom*). The corresponding CW EPR spectrum exhibits radical-type signals and some residual HS Fe(III). Closer inspection of the former (Fig. 5, *inset* to *bottom panel*) reveals that this signal is composite, displaying two features with a different saturation behavior (Fig. S2*A*). The most intense peak at *g* = 2.00 is in good agreement with the porphyrin π-cation radical observed in horseradish peroxidase (Fig. S2*C*) (27, 28). This intense signal disappears upon increase of the temperature above 10 K, leaving only the lower intense radical signal of the composite EPR part (Fig. S2*B*). A similar behavior is observed after H_2_O_2_ activation of DyP from *Thermomonospora curvata* (23). Multifrequency pulsed EPR experiments in combination with site-directed mutagenesis are currently ongoing to further elucidate the nature of all species in the resting and activated states.

### KpDyP forms a classical oxoiron(IV)-type Compound II

Next, we probed the enzymatic activity of KpDyP and investigated the spectral features of the involved redox intermediates of this heme peroxidase. WT KpDyP exhibits very poor overall peroxidase activities. Oxidation of bulky dyes like RB19, reactive black 5, indigo carmine, and acid red 183 was extremely slow. This also applies to conventional peroxidase substrates like guaiacol, pyrogallol, 3,3′,5,5′-tetrametylbenzidine (TMB), catechol, hydroquinones, tyrosine, and ascorbate (<0.5 units/mg) (not shown). Only ABTS was an exception, but also its oxidation is modest (∼40 units/mg). A search for alternative enzymatic activities (*e.g.* hydroxylation, dehalogenation, *N*-oxidation, etc.) did not yield any results.

**Figure 6.**
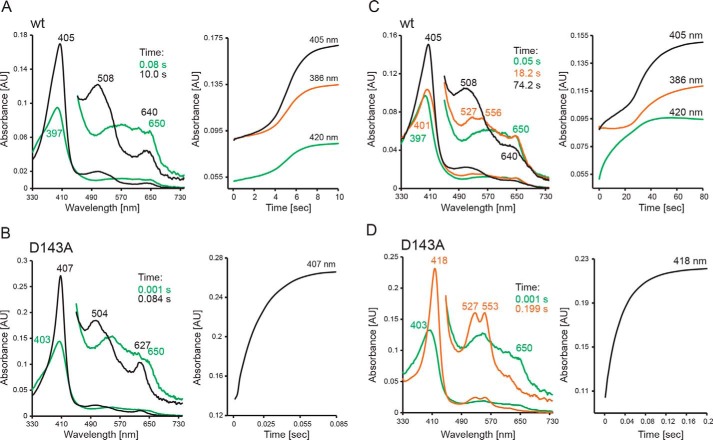
**Reaction of WT *Kp*DyP and D143A Compound I with thiocyanate and serotonin.** *A* and *C*, reaction of WT Compound I with thiocyanate (*A*) and serotonin (*C*) at pH 7.0, followed by the sequential stopped-flow mode. Compound I (*green spectrum*) was preformed by mixing 2 μm ferric *Kp*DyP with 4 μm H_2_O_2_ in 50 mm phosphate buffer, pH 7.0. After a 100-ms delay time, 1 mm thiocyanate (*A*) or 50 μm serotonin (*C*) was added. *B* and *D*, reaction of D143A Compound I with thiocyanate (*B*) and serotonin (*D*) at pH 7.0, followed by the sequential stopped-flow mode. Compound I (*green spectrum*) was preformed by mixing 2 μm ferric D143A with 1 mm μm H_2_O_2_ in 50 mm phosphate buffer, pH 7.0. After a 3000-ms delay time, 10 μm thiocyanate (*A*) or 1 mm serotonin (*C*) was added. Ferric spectra are shown in *black*, and oxoiron(IV)-type Compound II spectra are shown in *orange*. Relevant absorption maxima and time points of selection of the spectra are depicted.

Finally, we have probed the direct reaction between preformed Compound I of WT *Kp*DyP and the D143A variant with various putative one- and two-electron donors in the multimixing stopped-flow mode (Fig. 6). Regarding two-electron donors, chloride and bromide did not react with WT *Kp*DyP Compound I (formed with a 2-fold stoichiometric excess of H_2_O_2_), whereas iodide reacted only to a limited extent. Thiocyanate mediates the slow conversion of WT Compound I directly to the ferric state with isosbestic points at 356, 452, and 553 nm (Fig. 6*A*). The time traces monitored at 386 nm (isosbestic point between Compound I and Compound II; see below), 405 nm (Soret maximum of ferric state), and 420 nm (isosbestic point between Compound II and ferric state) are biphasic and show an initial lag phase followed by slow formation of the ferric state mediated by 1 mm thiocyanate. The lag phase may reflect cycling due to excess of H_2_O_2_, as its length increases with increasing excess of H_2_O_2_. No lag phase is observed when substoichiometric concentrations of H_2_O_2_ are used.

Elimination of Asp-143 significantly increased Compound I reactivity toward thiocyanate. (Fig. 6*B*). Compound I was formed with a 500-fold stoichiometric excess of H_2_O_2_. Upon reaction with thiocyanate, ferric D143A was formed rapidly and directly with clear isosbestic points at 432, 455, and 538 nm. Fig. 6*B* shows a representative time trace of the reaction between D143A Compound I and 10 μm thiocyanate. Due to the excess of H_2_O_2_ necessary for Compound I formation, exact determination of the apparent second-order rate constant was not possible (>10^6^ m^−1^ s^−1^).

Next we probed Compound I reduction by serotonin. It has to be noted that the addition of TMB leads to spectral changes comparable with those described for serotonin, whereas ascorbate and hydroquinone mediate only slow spectral conversions (*k*_obs_ > 0.5 s^−1^ with 1 mm). Tyrosine did not react at all (Fig. S3). Fig. 6*C* shows the reaction between WT *Kp*DyP Compound I (*green spectrum*) and 50 μm serotonin at pH 7.0. Compared with the direct two-electron reduction of Compound I to ferric *Kp*DyP, different spectral transitions were observed. During the very slow reaction, a redox intermediate accumulated after 15–20 s with spectral features reminiscent of oxoiron(IV)-type Compound II (absorbance bands at 527 nm and 556 nm; Fig. 6*C*, *orange spectrum*). Finally, this intermediate slowly disappeared, and the spectrum of ferric WT *Kp*DyP was formed (*black spectrum*). This conversion (Compound I → Compound II → ferric state) is also reflected by biphasic time traces monitored at 405 and 420 nm but not 386 nm (*i.e.* isosbestic point between Compound I and Compound II).

Again, the reactivity of the D143A variant Compound I with serotonin was several orders of magnitude higher at pH 7.0 compared with the WT enzyme (Fig. 6*D*). Conversion of D143A Compound I to an oxoiron(IV)-type Compound II (418, 527, and 553 nm; Fig. 6*D*, *orange spectrum*) was monophasic with clear isosbestic points at 397, 450, 502, and 572 nm. No further transitions were observed, probably due to excess of H_2_O_2_ and cycling of the variant. The apparent second-order rate constant *k*_app_ of Compound I reduction by serotonin was estimated to be ∼10^4^ m^−1^ s^−1^.

Finally, we have analyzed the serotonin-mediated reaction of Compound I by the ProK global analysis software (Applied Photophysics). Spectral simulation allowed isolation of the spectral features of ferric state, Compound I, and Compound II of WT *Kp*DyP at pH 7.0 and pH 10.0 during the time course of the reaction (Fig. S3). Data clearly support the stepwise formation of Compound I and oxoiron(IV)-type Compound II. However, in the WT enzyme (in contrast to the D143A variant), Compound II never fully accumulated. At pH 7.0, always a mixture of dominating Compound I, Compound II (<20%), and ferric state (<10%) is present. This ratio differs at pH 10 (50% Compound I, 40% Compound II, 10% ferric state) (Fig. S3, *C* and *D*). In any case, these findings clearly demonstrate that dye-decolorizing peroxidases of class B follow the classical peroxidase cycle with an oxoiron(IV)-type Compound II as redox intermediate.

### Heme active-site conformation is compromised by substitution of Arg-232

Finally, we elucidated the crystal structures of *Kp*DyP and the three variants at high resolution. The WT structure was solved by molecular replacement using the DyP structure from *V. cholerae* (*Vc*DyP). For the variants, the WT structure was used as a replacement model. The structures were refined to a resolution of 1.6 Å (WT), 1.27 Å (D143A), 1.86 Å (R232A), and 1.09 Å (D143A/R232A). Data collection and refinement statistics are given in Table 2, and a comparison of the 2*F_o_* − *F_c_* electron density maps contoured at σ = 1.5 of the active site is shown in Fig. S5.

**Table 2:** **Crystallization conditions, data collection, and refinement statistics for WT and *Kp*DyP variants** Values in parentheses are for the highest-resolution shell. r.m.s.d., root mean square deviation.

	WT *Kp*DyP	D143A	R232A	D143A/R232A
**Data collection**				
Wavelength	0.97949	0.966	0.966	0.966
Resolution range	52.22–1.6 (1.657–1.6)	52.68–1.27 (1.315–1.27)	57.07–1.86 (1.927–1.86)	48.16–1.09 (1.129–1.09)
Space group	P 1 2_1_ 1	P 1 2_1_ 1	P 6_5_ 2 2	P 1 2_1_ 1
Unit cell parameters				
*a*, *b*, *c* (Å)	50.52, 75.85, 75.64	50.76, 76.69, 76.14	91.16, 91.16, 247.82	50.62, 76.31, 75.94
α, β, γ (degrees)	90, 107.86, 90	90, 107.79, 90	90, 90, 120	90, 107.92, 90
Total reflections	215,756 (17,146)	346,778 (35,130)	332,227 (32,415)	641,984 (58,829)
Unique reflections	69,426 (6619)	142,004 (14,135)	51,833 (4908)	224,631 (22,440)
Multiplicity	3.1 (2.6)	2.4 (2.5)	6.4 (6.6)	2.9 (2.6)
Completeness (%)	96.86 (93.09)	97.21 (96.98)	99.26 (96.57)	98.45 (98.58)
Mean *I*/σ(*I*)	*F* (2.21)	16.61 (2.63)	12.11 (1.32)	10.82 (1.28)
Wilson *B*-factor	17.73	12.78	27.25	11.17

**Refinement**				
*R*_merge_	0.04149 (0.4457)	0.03012 (0.4241)	0.1134 (1.701)	0.04589 (0.691)
*R*_meas_	0.05004 (0.5549)	0.03792 (0.5324)	0.1235 (1.847)	0.05555 (0.8511)
*R*_pim_	0.02764 (0.3253)	0.02269 (0.3181)	0.04821 (0.7093)	0.0309 (0.4891)
CC1/2	0.999 (0.802)	0.999 (0.794)	0.998 (0.674)	0.999 (0.574)
CC*	1 (0.944)	1 (0.941)	1 (0.897)	1 (0.854)
Reflections used in refinement	69,394 (6619)	141,989 (14134)	51,706 (4905)	224,597 (22,434)
Reflections used for *R*_free_	3418 (335)	6954 (652)	2566 (247)	11,225 (1135)
*R*_work_	0.1281 (0.1757)	0.1147 (0.0.1760)	0.1677 (0.2959)	0.1246 (0.2533)
*R*_free_	0.1668 (0.2198)	0.1406 (0.2212)	0.2074 (0.3242)	0.1495 (0.2747)
CC(work)	0.973 (0.935)	0.977 (0.939)	0.969 (0.797)	0.978 (0.802)
CC(free)	0.966 (0.893)	0.973 (0.909)	0.950 (0.733)	0.972 (0.762)
No. of non-hydrogen atoms	5326	5682	5219	5817
Macromolecules	4689	4781	4644	4928
Ligands	112	106	87	112
Solvent	525	795	488	777
Protein residues	596	602	598	602
r.m.s.d. (bonds)	0.007	0.01	0.004	0.010
r.m.s.d. (angles)	0.97	1.08	0.73	1.11
Ramachandran favored (%)	98.31	98.83	97.98	98.49
Ramachandran allowed (%)	1.69	1.17	1.85	1.51
Ramachandran outliers (%)	0	0	0.17	0
Rotamer outliers (%)	0.82	0.4	0.62	0.20
Clashscore	1.37	1.17	0.97	1.70
Average *B*-factor	21.26	18.06	33.5	16.61
Macromolecules	19.93	15.81	32.81	14.51
Ligands	18.2	12.18	31.83	12.01
Solvent	33.75	32.34	40.35	30.61

**Figure 7.**
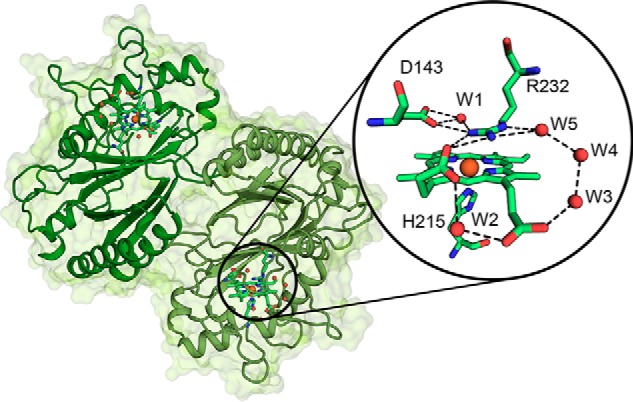
**Overall and active-site crystal structure of WT *Kp*DyP.** The dimeric structure of *Kp*DyP is shown as a *cartoon* (*green*). Heme prosthetic groups are depicted as *sticks*, and the heme iron is shown as an *orange sphere*. The *round inset* shows a detailed view of the active-site amino acid residues Asp-143 and Arg-232 (distal) and His-215 (proximal) and the heme *b* moiety as a *stick representation*. Hydrogen-bonding networks involving the distal residues and the heme *b* propionates are shown as *dashed lines*, and water molecules (*W*) and heme iron are depicted as *red* and *orange spheres*, respectively.

All structures exhibit a dimeric ferredoxin-like fold typical for dye-decolorizing peroxidases, composed of antiparallel β-sheets with four β-strands connected by α-helices. Fig. 7 depicts the structure of WT *Kp*DyP. For all figures, we use the chain A subunit, but both subunits are in good agreement with respect to amino acid position, ligands, and intramolecular distances if not noted otherwise. One heme *b* cofactor is present per subunit, with His-215 acting as proximal ligand at a distance of 2.2 Å between the heme iron and Nϵ. The proximal ligand is hydrogen-bonded to Asp-268.

At the distal heme side, a pronounced hydrogen-bonding network is observed, including the proposed catalytic residues Asp-143 and Arg-232, water molecules (W1, W3, W4, and W5), and propionate p6. Propionate p6 and p7 are connected by hydrogen bonding via the water molecule W2. Importantly, a salt bridge between Arg-232 and propionate p7 stabilizes the distal architecture. As already observed in other B-class DyP structures, there are two access channels to the heme. The distal access channel to the active-site pocket composed of Asp-143, Arg-232, Phe-248, Leu-246, and Ser-234 is narrow with a calculated bottle neck radius of 1.2 Å (calculated by CAVER). The second access from the surface is via the solvent-exposed propionate p6 (Fig. 7).

**Figure 8.**
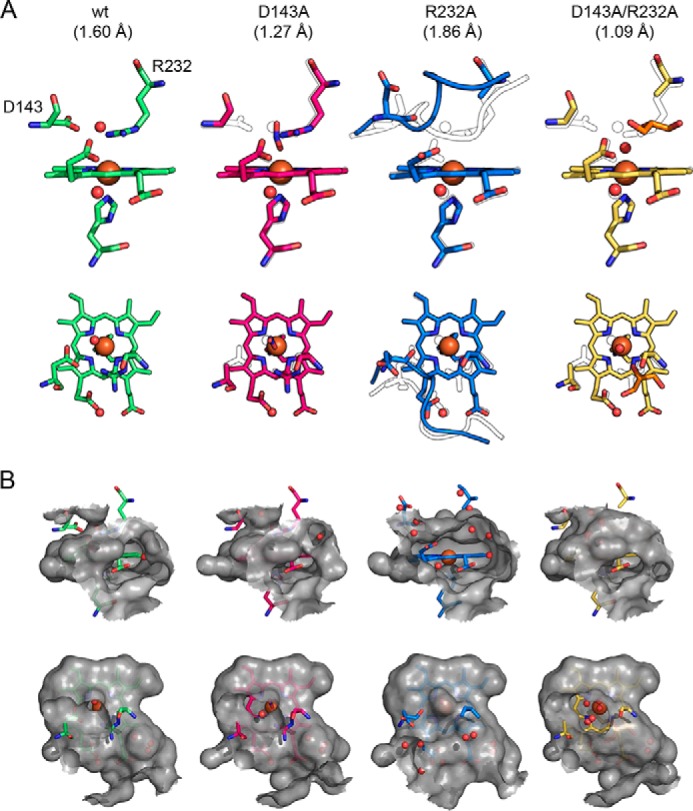
**Comparison of the active-site architecture of WT *Kp*DyP and the variants D143A, R232A, and D143A/R232A.** *A*, representation of the active-site residues Asp-143, Arg-232, and His-215 and the heme *b* moiety shown from the *front* (*first row*) and *top* (*second row*). Relevant water molecules are shown as *red spheres*, nitrite (D143A structure) is shown in *blue*, and glycerol (D132A/R232A structure) is shown in *orange*. In all mutant structures, the WT structure is depicted as a *black outline* for comparison. Additionally, the backbones of residues 141–148 are shown in the R232A structure together with the respective WT conformation (*black outline*). *B*, surface of the active site and access channels shown in a 6-Å radius from the heme iron *colored* in *gray* (*semitransparent*), shown from the *front* (*first row*) to visualize the surface-accessible propionates and from the *top* (*second row*) to show the distal access channel.

Substitution of the distal residues Asp-143 and Arg-232 did not lead to significant changes in the overall fold of the enzyme. Exchange of Asp-143 by alanine does not change the active-site architecture (Fig. 8 and Fig. S5). However, in D143A, the distal access channel is significantly bigger, whereas the surface exposure of propionate p6 remains similar to the WT protein (Fig. 8). In the structures of D143A, a molecule of unknown origin (refined as nitrite) was repeatedly found ligated to the heme iron at a distance of 2 Å. However, the presence of this ligand had no impact on the conformation of the distal residues.

In contrast to D143A, the R232A variant shows significant changes in the active-site conformation, with residues 142–148 found in a completely different orientation compared with the WT structure (Fig. S4). These structural alterations might cause the different space group of the crystal structure of R232A compared with WT *Kp*DyP despite very similar crystallization conditions (Table 2). The residues 142–148 form a loop that amounts to 50% of the solvent-exposed active site. The new loop conformation in R232A is stabilized on one side through two hydrogen bonds formed between Asp-143 and the nearby Arg-131 and one between Thr-135 and Arg-232 as well as on the other side by the change of Pro-148 from a trans- to a cis-conformation. In the crystal structure of R232A, Asp-143 is pointing away from the heme *b* cofactor, and the distance between the carboxylate oxygen and the heme iron increases from 5.0 to 8.9 Å. In addition, reorientation of the loop leads to the loss of the distal access channel, whereas both heme *b* propionates are completely solvent-exposed. Notably, the orientation of the propionate p7 also changes, most likely due to the missing salt bridge. The closest distal water is 4.96 Å away from the heme iron in chain A and 4.1 Å in chain B.

Interestingly, the crystal structure of the variant D143A/R232A is similar to the WT structure. Simultaneous substitution of Asp-143 and Arg-232 by alanine leads to increased accessibility of the active site through the distal access channel. It is noteworthy that crystals could only be obtained with the addition of 5% glycerol. As a consequence, a glycerol molecule occupies the position of substituted Arg-232 in D143A/R232A and is extensively involved in hydrogen bonding with the propionate p7 and surrounding water molecules. We propose that this glycerol replaces Arg-232 in its role to stabilize the WT-like conformation of the loop comprising residues 142–148. In the double variant, the distal iron coordination position is occupied by a water molecule at a distance of 2.2 Å.

## Discussion

In the present study, we have aimed to elucidate the mechanism of Compound I formation and reduction by comparison of WT *Kp*DyP and variants having the conserved putative catalytic distal residues Asp-143 and Arg-232 exchanged by alanine. Importantly, the recombinantly produced variants D143A, R232A, and D143A/R232A showed WT-like ECD spectra in the far- and near-UV region and pH-dependent thermal stabilities similar to, albeit slightly lower than, that of the WT protein. Moreover, the high-resolution crystal structures of WT *Kp*DyP and the investigated variants showed that the performed mutations did not alter the overall fold, which is similar to other B-class DyPs (6, 10–11). Together, these findings demonstrated that between pH 4.5 and pH 9.0, all four proteins were correctly folded and thus allowed comparative kinetic studies within this pH regime.

In all DyPs, the prosthetic group (*i.e.* heme *b*) is coordinated by a proximal histidine (His-215), which is hydrogen-bonded with a fully conserved aspartate (Asp-268). The negative standard reduction potential *E*°′ of the Fe(III)/Fe(II) couple of −350 mV clearly suggests a pronounced imidazolate character of His-215 in *Kp*DyP. In other B-class DyPs, the corresponding *E*°′ values were reported to be more positive (*e.g.* −260 mV in *Pp*DyP (8) and −290 mV in *El*DyP (11)). Interestingly, the impact of the introduced substitutions is relatively small. Elimination of a negative charge by exchange of Asp-143 by alanine should increase *E*°′(Fe(III)/Fe(II)). However, in D143A, the small effect (+20 mV) seems to reflect the increase in exposure of the heme iron to solvent compared with WT *Kp*DyP (Fig. 8). By contrast, elimination of a positive charge in the heme cavity should decrease the reduction potential. Here, substitution of Arg-232 by alanine does not significantly influence the *E*°′ (−3 mV), which indicates that the loss of Arg-232 is compensated by loss of the negatively charged carboxylate group of Asp-143 that points away from the heme iron in R232A (Fig. 8). In any case, the ferric state is highly stabilized both in WT *Kp*DyP and the variants.

We have demonstrated that the distal hydrogen-bonding network including Asp-143, Arg-232, and several water molecules (W1–W5) as well as the propionate side chains p6 and p7 of WT *Kp*DyP is stable between pH 4.5 and pH 10.0, as is reflected by almost identical HS spectra determined by both UV-visible and EPR spectroscopy. No alkaline transition was observed, suggesting identical distances between Asp-143, Arg-232, W1, and the heme iron in this pH regime. The variant D143A is unique in showing an alkaline transition (p*K_a_* = 8.6) to a HS hydroxo state. For each *Kp*DyP variant, EPR revealed the presence of multiple HS species with varying rhombicity, as reflected by the different *E/D* values (Fig. 1*B*). Co-existence of several HS species is not uncommon for peroxidases (Table 1). Different HS forms were detected for catalase-peroxidase (KatG) from *Mycobacterium tuberculosis*, and these forms appeared to be pH-dependent (29). The EPR spectra of chlorite dismutase from *Magnetospirillum* sp. showed different HS and LS features that were influenced by the purification method (30), NaCl excess (30), pH, and aging (31). On the contrary, only one HS species was found in the dimeric chlorite dismutase from *Cyanothece* sp. PCC7425 (*C*Cld) (32). CW EPR studies on the A-class DyP of *T. curvata* and a fungal DyP of *Auricularia auricula-judae* also show a pH dependence of the HS forms and a heme-pocket heterogeneity, respectively (22, 23). These proteins display, however, a much lower rhombicity of the dominant species than WT *Kp*DyP (Table 1) and the A-class DyP from *R. jostii RHA1* (6).

The crystal structures of WT *Kp*DyP revealed two heme access channels. One narrow channel leads perpendicularly to the distal heme pocket, and its small bottleneck radius of 1.2 Å suggests that it only allows access of H_2_O_2_ or small ligands like cyanide. This channel also exists in B-class DyPs of *R. jostii* RHA1, *Vc*DyP, and *El*DyP with reported bottleneck radii of 2–3 Å. The second access channel is relatively open and leads to the solvent-exposed propionate p6. Here, principally, potential substrates could bind and deliver electrons. Exchange of Asp-143 by alanine kept the heme cavity architecture WT-like but significantly increased the distal channel diameter, whereas substitution of Arg-232 by alanine induced a collapse and reorganization of the heme cavity leading to loss of the distal heme access channel. Additionally, in the crystal structure of R232A, the side chain of Asp-143 points away from the heme iron. These data indicate that it is not reliable to discuss the role of Arg-232 in catalysis of Compound I formation in *Kp*DyP based on the kinetic data of R232A. It remains open whether less drastic substitutions of Arg-232 do not result in collapse of the heme pocket of *Kp*DyP.

Oxidation of ferric WT *Kp*DyP by hydrogen peroxide is extremely fast (∼6.2 × 10^6^ m^−1^ s^−1^), monophasic, and pH-independent between pH 4 and pH 9. CW EPR spectroscopic data recorded at 2.5 K revealed the electronic structure of Compound I to be an oxoiron(IV) porphyrin radical species. This is also reflected by the monitored UV-visible spectral changes (*i.e.* 50% hypochromicity in the Soret region) during the reaction between ferric *Kp*DyP and equimolar H_2_O_2_.

Compound I formation in heme peroxidases requires an initial interaction (“ligand binding”) of H_2_O_2_ with the ferric heme iron (*k*_1_) (33). This is followed by deprotonation of H_2_O_2_ and binding of the resulting anion to the ferric heme iron (*i.e.* formation of so-called Compound 0) (*k*_2_). Compound 0 formation is followed by rapid heterolytic cleavage of the peroxide bond (*k*_3_), thereby forming Compound I and water. In two heme peroxidase superfamilies (4) (*i.e.* peroxidase-catalase and peroxidase-cyclooxygenase superfamilies), a fully conserved distal His–Arg pair supports Compound I formation. The distal His acts as acid–base catalyst and deprotonates the incoming H_2_O_2_ and protonates the distal oxygen during heterolytic cleavage. Additionally, the negatively charged outer oxygen atom seems to be transiently stabilized by the guanidinium group of the conserved arginine (15). The rates of Compound I formation in these peroxidases are similar (1–5 × 10^7^ m^−1^ s^−1^) (34) and slightly higher compared with B-class DyPs. Typically, in these peroxidases, heterolytic cleavage of the O–O bond in Compound 0 is significantly faster than Compound 0 formation (*k*_3_ ≫ *k*_2_). As a consequence, Compound 0 is difficult to trap.

**Figure 9.**
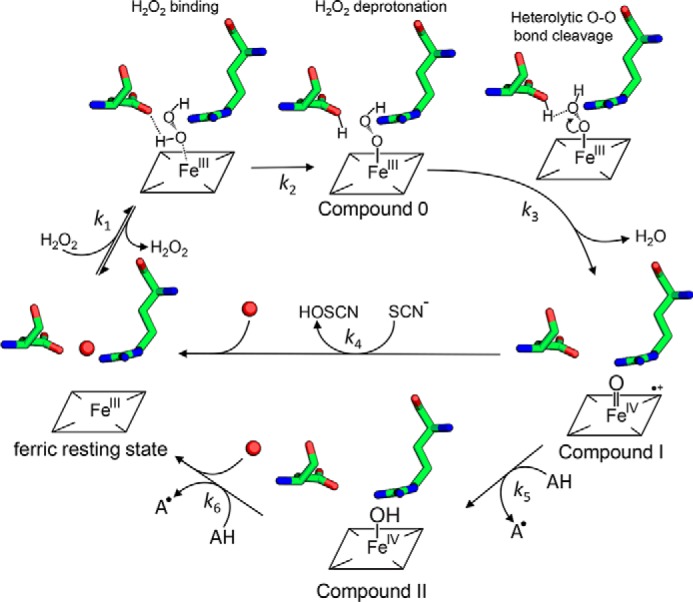
**Proposed reaction mechanism for Compound 0, Compound I, and Compound II formation in B-class DyPs.** Hydrogen peroxide enters the narrow distal heme access channel, binds (*k*_1_), and is deprotonated by Asp-143 (Compound 0 formation). Heterolytic cleavage of the O–O bond (*k*_3_) forms Compound I (oxoiron(IV) porphyrin radical) (*k*_3_), which is reduced by either two-electron donors like thiocyanate (^−^*SCN*) directly to the ferric resting state, thereby producing hypothiocyanite (*HOSCN*) (*k*_4_), or by one-electron donors like serotonin (*AH*) to Compound II (oxoiron(IV) species) (*k*_5_), thereby producing the corresponding radical (*A*^•^). Finally, a second one-electron donor (*AH*) reduces Compound II to the ferric resting state (*k*_6_).

Our data support a similar mechanism for Compound I formation in B-class DyPs (Fig. 9) with distal Asp-143 acting as acid–base catalyst. The observed pH independence of Compound I formation within the range pH 4.0–9.0 indicates that the p*K_a_* of the side chain of Asp-143 is far below 4.0 because its conjugated base is stabilized through the proximity to the guanidinium group of Arg-232. In the absence of Asp-143, the apparent bimolecular rate constant of Compound I formation is reduced by 3 orders of magnitude at pH 7.0, and the pH independence disappears. Interestingly, whereas a similar impact was observed in *E. lignolyticus* (11) (∼2 × 10^2^ m^−1^ s^−1^), the same substitution had only a minor impact on Compound I formation in *R. jostii* (7) (9.4 × 10^4^ m^−1^ s^−1^ compared with 2 × 10^5^ m^−1^ s^−1^ in the WT protein). However, in A- and C/D-class DyPs, it was demonstrated that the conserved distal aspartate is essential in Compound I formation (3, 18).

In the *Kp*DyP variant D143A, the rate of Compound I formation increases with increasing pH, suggesting the assistance of hydroxide ions in H_2_O_2_ deprotonation. This scenario is also supported by the kinetics of cyanide binding to ferric WT *Kp*DyP and to the variant D143A. Cyanide, which at most biological pH levels is protonated (p*K_a_* = 9.14), must also be deprotonated before binding to ferric heme proteins. Thus cyanide complex formation mirrors H_2_O_2_-mediated formation of Compound 0 in peroxidases. Typically, the apparent bimolecular rate constant of cyanide complex formation of WT *Kp*DyP is pH-independent, whereas cyanide binding to D143A increases by 1 order of magnitude per pH step between pH 5.0 and pH 9.0. Consequently, our data suggest that Compound 0 formation (but not heterolytic cleavage of the O–O bond in Compound 0) is the rate-limiting step in B-class DyPs. This was also proposed for Compound I formation in *El*DyP based on studies on solvent isotope and viscosity effects (11).

Because the distal heme cavity collapsed upon elimination of Arg-232 by alanine (Fig. 8*A* and Fig. S4*A*), we cannot provide clear evidence of its role in heterolytic cleavage of hydrogen peroxide. In Compound 0, the hydroperoxyl anion must replace W1 (Fig. 7). As a consequence, the positively charged guanidinium group comes close to the distal oxygen atom and will electrostatically promote the heterolysis of the O–O bond to form Compound I. The importance of this transient interaction is difficult to evaluate because the heme architecture collapsed in R232A.

It has to be noted that in the absence of both Asp-143 and Arg-232, Compound I formation still occurred, albeit more than 3 orders of magnitude slower. Substitution of the catalytically relevant histidine and arginine in horseradish peroxidase decreases the rate of Compound I formation by a factor of 10^6^ and 10^4^, respectively, but does not abolish it (*k*_app_ ∼20 m^−1^ s^−1^ and 3 × 10^3^ m^−1^ s^−1^) (35, 36). The same was observed for cytochrome *c* peroxidase, where similar mutations reduced Compound I formation by a factor of 10^5^ (*k*_app_ ∼300 m^−1^ s^−1^) and 10^2^ (*k*_app_ ∼3 × m^−1^ s^−1^) (37, 38), respectively. Slow Compound I formation in the absence of potential distal catalytic residues has also been described recently in the structurally and evolutionarily related HemQ from *Listeria monocytogenes*, which catalyzes the H_2_O_2_-mediated decarboxylation of coproheme to heme *b.* However, the mechanism of Compound I formation in HemQ is unknown (39).

In any case, Compound I of *Kp*DyP is very stable, as reflected by the absence of spectral shifts within minutes. No internal electron transfer reactions occur, which in other heme peroxidases lead to Compound I* formation with typical oxoiron(IV)-type Compound II spectrum (according to Reaction 3) (40). Compound I stability is also reflected by the very negative reduction potential of the Fe(III)/Fe(II) couple (*i.e.* −350 mV). Typically, *E*°′ values of the Fe(III)/Fe(II) couple strictly correlate with *E*°′ values of the catalytically relevant redox couples Compound I/Compound II as well as Compound I/ferric state in heme peroxidases (41–44). The more positive the *E*°′ value of the redox couple Fe(III)/Fe(II) is (ranging from −350 to +50 mV) (40), the more unstable and reactive is Compound I of the respective peroxidase.

In the case of WT *Kp*DyP, the extraordinary stability of Compound I strictly correlates with very poor peroxidase activities. Within physiological (*i.e.* cytosolic) pH regimes (pH 5.0–8.0), Compound I of this cytosolic peroxidase did not react with two-electron donors like chloride or bromide and to a limited extent with iodide. Only thiocyanate induced a direct but very slow conversion to the ferric resting state (*k*_4_). Halides and thiocyanate typically bind at the distal heme side of peroxidases close to the oxoiron(IV) oxygen of Compound I (45). Importantly, the corresponding two-electron reduction potential of thiocyanate is low (560 mV) (46). Thus, the poor reactivity of *Kp*DyP Compound I cannot be attributed to thermodynamic reasons only. In addition, the restricted accessibility via the small distal access channel might contribute to the observed poor reaction rates. This is supported by the fact that in D143A Compound I, the reduction rate of thiocyanate was significantly enhanced due to both higher reactivity and unhampered access, as indicated by the crystal structure of D143A.

Finally, for the first time, we could identify spectroscopically the formation of an oxoiron(IV)-type Compound II in a B-class DyP with typical absorbance maxima at 418, 527, and 553 nm, when Compound I was mixed with serotonin as a one-electron donor. This is in contrast to other reports on redox intermediates of the peroxidase cycle of B-class DyPs (11). Due to very poor activities (or spectral interference) observed with most conventional peroxidase substrates, serotonin was selected. Due to its low standard reduction potential of 650 mV (47), oxidation of serotonin by Compound I (*k*_5_) is typically very fast with all so far studied heme peroxidases from other superfamilies (48). The fact that in B-class DyPs serotonin is a poor electron donor indicates that neither access channel provides optimal binding and oxidation sites for aromatic substrates. Around neutral pH, Compound II did not fully accumulate during steady-state oxidation of serotonin but was clearly visible by the characteristic absorbance bands at 527 and 553 nm. This suggests similar rates of serotonin-mediated Compound I (*k*_5_) and Compound II reduction (*k*_6_). Interestingly, and similarly to SCN^−^ oxidation (*k*_4_), D143A Compound I reduction was several orders of magnitude faster compared with the WT enzyme. Both the WT and D143A crystal structures show an almost identical heme access via the broad channel leading to propionate p6. However, elimination of Asp-143 opens the entrance to the distal cavity, probably facilitating substrate access to the heme moiety. In any case, during turnover of D143A, Compound II accumulated, suggesting that Compound I reduction rates are significantly higher than Compound II reduction rates (*k*_5_ > *k*_6_) in this variant.

In conclusion, heterolytic cleavage of hydrogen peroxide or binding of cyanide by *Kp*DyP is pH-independent, very fast, and initiated by rate-limiting Compound 0 formation. The fully conserved distal aspartate is responsible for deprotonation of H_2_O_2_, whereas arginine 232 is important for maintaining the distal heme cavity architecture and might support heterolysis of the O–O bond by transiently and electrostatically interacting with the negatively charged distal oxygen atom. Compound I, an oxoiron(IV) porphyrin radical species, is very stable and only very slowly reacts with two- and one-electron donors. Reduction of Compound I by thiocyanate leads directly to ferric *Kp*DyP, whereas reduction by serotonin forms oxoiron(IV)-type Compound II as redox intermediate. Elimination of Asp-143 significantly enhances the reactivity of Compound I with both one- and two-electron donors. Based on our data, we challenge the proposal that peroxidase activity toward conventional aromatic substrates or dyes is related to the physiological role of B-class DyPs. At the moment, we are not able to answer the question about *in vivo* electron donor(s) of Compound I of B-class DyPs. A study on potential cytosolic interaction partners is in progress.

## Experimental procedures

### Cloning, site-directed mutagenesis, expression, and purification of KpDyP and its variants

The codon-optimized DNA for *Kp*DyP was purchased from ATUM (Newark, CA) and cloned in frame into pGEX-6P1 using a Gibson Assembly kit (New England Biolabs, Ipswich, MA) and primers 5′-TCAGTCACGATGCGGCCGCTCGATCAACCGAGCGCTTGAATACGCTC-3′ and 5′-TCCAGGGGCCCCTGGGAATGAGCCAAGTACAGAGCGGT-3′ for protein production using the GSTrap system. The variants were created by site-directed mutagenesis using the QuikChange Lightning kit (Agilent Technologies, Santa Clara, CA) according to the manufacturer's description and primers 5′-GTCTGGTTTCGTGGCCGGCACCGAGAACC-3′ and 5′-GGTTCTCGGTGCCGGCCACGAAACCAGAC-3′ for D143A,5′-GGCCTGAAGATTGTGGCCCAGAGCTTGCCGTA-3′ and 5′-TACGGCAAGCTCTGGGCCACAATCTTCAGGCC-3′ for R232A, and a combination of both primer sets for the double variant.

The recombinant proteins were expressed heterologously in *E. coli* BL21 (DE3) carrying the pLysS plasmid (Merck/Novagen, Darmstadt, Germany) in Luria broth supplemented with ampicillin and chloramphenicol. Cells were incubated at 37 °C and 180 rpm until *A*_600_ = 0.8, and then the cultivation temperature was reduced to 16 °C. Protein expression was induced after 1 h using 0.5 mm isopropyl-β-d-thiogalactopyranoside (final concentration), and after 16 h of growth at 16 °C, cells were harvested by centrifugation (4500 rpm, 25 min, 4 °C) and stored at −30 °C.

For protein purification, cell pellets were thawed in lysis buffer (50 mm Tris-HCl, pH 7.3, 150 mm NaCl, 0.1% Triton X-100) with 1 mm phenylmethylsulfonyl fluoride and 100 μm hemin (final concentration). Cells were lysed by sonication (Vibra Cell, Sonics & Materials Inc., Danbury, CT; 50% power, 3 × 60 s) and clarified by centrifugation (18,000 rpm, 25 min, 4 °C). The cleared lysate was filtered (0.45 μm; Millipore) and loaded onto a GSTrap HP 5-ml (GE Healthcare) column pre-equilibrated with binding buffer (50 mm Tris-HCl, pH 7.3, 150 mm NaCl). The loaded columns were washed with binding buffer and pre-equilibrated with cleavage buffer (50 mm Tris-HCl, pH 7.0, 150 mm NaCl). For on-column cleavage, columns were manually loaded with GST-tagged HRV 3C protease diluted in 5 ml of cleavage buffer and incubated overnight at 4 °C. Elution was performed with 5 ml of cleavage buffer. The eluted protein solution was concentrated using 50-ml Amicon centrifugation units (30-kDa cutoff). The concentrated protein was applied to a HiLoad 16/60 Superdex 200 prep grade column (GE Healthcare), pre-equilibrated with 50 mm phosphate buffer, pH 7.0, and collected in 500-μl fractions. All fractions with a Reinheitszahl value > 1.9 for the WT protein and the D143A variant and 2.5 for the variants R232A and D143A/R232A were collected, concentrated to > 500 μm, aliquoted, and stored at −80 °C.

### UV-visible and ECD

All UV-visible spectra were measured at room temperature on a Hitachi U-3900 spectrometer. For each measurement, 1000 μl of *Kp*DyP with a concentration of 10 μm were prepared in 50 mm phosphate buffer (pH 7.0) and used for UV-visible analysis. For all experiments involving a comparison between pH 4.0 and 8.0, 50 mm phosphate citrate buffer was used, and between pH 8.0 and 10.0 50 mm glycine NaOH was used.

For secondary structure prediction, ECD spectra were taken in the far-UV (180–260 nm) using Chirascan from Applied Photophysics (Leatherhead, UK), the bandwidth was set to 3 mm, path length was 1 mm, and scan speed was 10 s nm^−1^. The instrument was flushed with nitrogen at a flow rate of 5 liters min^−1^ and allowed simultaneous UV-visible and ECD monitoring. Samples (400 μl of enzyme solution (10 μm) in 5 mm phosphate buffer (pH 7.0) were measured at room temperature. For ECD spectra in the near-UV and visible ranges (260–450 nm), the bandwidth was set to 3 mm, the path length was 10 mm, and the scan speed was 5 s nm^−1^. Samples consisted of 1 ml of enzyme solution (10 μm) in 50 mm phosphate buffer (pH 7.0).

### EPR

For EPR spectroscopic studies, 500 μm protein was prepared in 50 mm phosphate buffer, pH 7.0. In all cases, 30% glycerol was added as a cryoprotectant. Typically, 100 μl was inserted in a quartz EPR tube and flash-frozen in liquid N_2_. In addition, the WT protein was activated by adding a 5-fold molar excess of H_2_O_2_ to generate Compound I. The EPR sample tubes were vacuum-pumped to 1 millibar during the experiments to remove excess of paramagnetic dioxygen. X-band CW EPR experiments were performed on a Bruker ESP300E spectrometer operating at a microwave frequency of ∼9.44 GHz equipped with a liquid-helium cryostat (Oxford Inc.) to enable temperatures from 2.5 K up to room temperature. Calibration of the magnetic field was done using a Bruker ER035M NMR gaussmeter.

All spectra of the ferric proteins were recorded at 4 K under nonsaturating conditions at 1-milliwatt microwave power and 0.5-mT modulation amplitude. The detailed EPR spectrum of the organic radical in the resting state protein was recorded at 80 K using 0.1-milliwatt microwave power and 1-mT modulation amplitude. The EPR spectrum of compound I was recorded at 2.5 K using 100-microwatt microwave power and 0.1-mT modulation amplitude. In all cases, the modulation frequency was 100 kHz. Simulation of the experimental spectra was done using the Matlab (MathWorks, Natick, MA)-based software Easyspin (49).

### Differential scanning calorimetry

Temperature-induced pH-dependent protein unfolding studies were performed using DSC. All samples were analyzed between 20 and 100 °C. Samples were heated at 60 psi (4.136 bar) with a rate of 60 °C h^−1^ cell pressure using a VP-capillary DSC microcalorimeter from Microcal (cell volume: 137 μl), equipped with an autosampler for 96-well plates. All samples consisted of 10 μm enzyme in 50 mm phosphate–citrate buffer, pH 3.0–7.5 (WT) and pH 4.5–7.5 (variants) and were kept at 4 °C before measurement. Collected data were corrected for buffer baseline, normalized for protein concentration, and analyzed using the Microcal Origin software package.

### Spectroelectrochemistry

The standard reduction potential, *E*°′, of the Fe(III)/Fe(II) couple was determined using a homemade OTTLE cell (41, 50–52). The three-electrode configuration consisted of a gold minigrid working electrode (Buckbee-Mears), a homemade Ag/AgCl/KCl_sat_ microreference electrode, separated from the working solution by a Vycor set, and a platinum wire as the counter electrode (41, 50–52). The reference electrode was calibrated against a saturated calomel (HgCl) electrode before each set of measurements. All potentials are referenced to the standard hydrogen electrode (+242 mV). Potentials were applied across the OTTLE cell with an Amel model 2053 potentiostat/galvanostat. A constant temperature was maintained by a circulating water bath, and the OTTLE cell temperature was monitored with a copper-costan microthermocouple. UV-visible spectra were recorded using a Varian Cary C50 spectrophotometer (Agilent Technologies, Santa Clara, CA). The OTTLE cell was flushed with argon gas to establish an oxygen-free environment in the cell. Spectroelectrochemical titrations were performed with 15 μm WT *Kp*DyP or mutants in 100 mm sodium phosphate buffer (pH 7.0) plus 100 mm NaCl. Additionally, 25 μm methyl viologen and 1 μm lumiflavin 3-acetate, methylene blue, phenazine methosulfate, and indigo disulfonate were used as redox mediators.

### Steady-state and transient-state kinetics

Substrate specificity was determined by following the increase or decrease of absorbance at specified wavelengths for 120 s using a stirred cuvette and a scanning photometer (Hitachi U-3900, Tokyo, Japan). A typical reaction consisted of 1 ml of 50 mm phosphate–citrate buffer, pH 4.0 to pH 7.0, with 100 μm, 1 mm, or 10 mm substrate, 500 μm H_2_O_2_, and 200 nm enzyme. All reactions were carried out in triplicates.

All transient-state experiments were conducted with a stopped-flow apparatus (SX-18MV or pi-star fitted equipped with diode array detector or a monochromator) from Applied Photophysics. The optical quartz cell with a path length of 10 mm had a volume of 20 μl. The fastest time for mixing was 0.68 ms, and all measurements were performed at 25 °C. For single wavelength measurements, a minimum of three runs were performed for each ligand or substrate concentration. Typically, in studies of the kinetics of formation of the *Kp*DyP–cyanide complex, 2 μm ferric protein in 50 mm phosphate buffer, pH 7.0, was mixed with at least a 10-fold excess of cyanide in the same buffer. The reaction was monitored by following the increase of absorbance at 420 nm. For studies on the pH dependence of cyanide binding, 2 μm enzyme in 5 mm phosphate citrate buffer, pH 7.0, was mixed with at least 10-fold excess of cyanide in 100 mm phosphate–citrate buffer, pH 3.0–8.0, or 100 mm glycine-NaOH buffer, pH 8.0–10.0.

In addition to elucidation of the kinetics of cyanide binding to ferric B-class DyP, the thermodynamics of the low-spin complex formation was investigated. In detail, for ligand titration, 1 ml of 5–10 μm protein was prepared in 50 mm phosphate buffer, pH 7.0. The titrations were performed in a stirred cuvette at 25 °C and monitored by UV-visible absorption spectroscopy between 250 and 700 nm using a scanning photometer (Hitachi U-3900, Tokyo, Japan). Cyanide was added stepwise from a 1 m stock solution. All spectra were corrected for the dilution and smoothed using the Savitzky–Golay smoothing algorithm provided by the Hitachi U-3900 software package.

For studying the kinetics of Compound I formation, the conventional stopped-flow mode was used. In a typical experiment, 2 μm ferric enzyme in 50 mm phosphate buffer, pH 7.0, was mixed with at least a 10-fold excess of H_2_O_2_ in the same buffer. The pH dependence of this reaction was measured with 2 μm enzyme in 5 mm phosphate citrate buffer, pH 7.0, mixed with at least 10-fold excess of hydrogen peroxide in 100 mm phosphate citrate buffer, pH 3.0–8.0, or 100 mm glycine NaOH buffer, pH 8.0–10.0.

For studying the reactivity of WT *Kp*DyP and the variant D143A Compound I with the two-electron donor thiocyanate and the one-electron donor serotonin, the sequential stopped-flow mode was used. In detail, Compound I was formed by mixing 2 μm ferric WT *Kp*DyP with a 2-fold stoichiometric excess of hydrogen peroxide in 50 mm phosphate buffer, pH 7.0. After a delay time of 100 ms, Compound I was mixed with varying concentrations of thiocyanate or serotonin. In the case of the D143A variant, Compound I was preformed by mixing 2 μm D143A with a 500-fold excess of H_2_O_2_, and the delay time was 3000 ms.

### Crystallization and structure determination

All enzymes crystallized in optimized conditions of SG1 Screen HT-96 (Molecular Dimensions, Newmarket, UK) well C9. Crystallization experiments were performed using SWISSCI MRC three-well crystallization plates (Molecular Dimensions), adopting the vapor diffusion method. Crystallization drops were set using a Mosquito LCP (TTP Labtech, Melbourn Science Park, Melbourn, UK). All protein stocks were prepared in a final concentration of 8.25 mg ml^−1^ in 50 mm phosphate buffer, pH 7.0. The seed solution was prepared by collecting all crystals of a single well in 50 μl of 50 mm phosphate buffer, pH 7.0, containing 10 1.0-mm glass beads followed by pulverization through 4 × 1-min vortexing at full speed. The initial seed stock was diluted, depending on the amount of crystals used. The reservoir was filled with 40 μl of crystallization solution. Crystallization drops were set up with enzyme/seed/crystallization solution ratios of 100:100:200, 200:100:200, and 300:100:200 nl. Crystallization plates were stored at 22 °C.

WT *Kp*DyP crystallized in 23% (w/v) PEG 3350, 0.1 m MgCl_2_, 0.1 m Tris-HCl, pH 8.5; the D143A variant in 23% (w/v) PEG 3350, 0.1 m MgCl_2_, 0.1 m Tris-HCl, pH 8.5; R232A in 29% (w/v) PEG 3350, 0.15 m MgCl_2_, 0.1 m Tris-HCl, pH 8.5; and D143A/R232A in 23% (w/v) PEG 3350, 0.1 m MgCl_2_, 0.1 m Tris-HCl, pH 8.5, 5% (w/v) glycerol. For cryoprotection, the crystallization conditions were supplemented with 25% (w/v) glycerol. All crystals were harvested using cryoloops and flash-vitrified in liquid nitrogen.

Data sets were collected at beamline ID30A-1 (53) of the European Synchrotron Radiation Facility (Grenoble, France) at 100 K using a PILATUS3 2 M detector or at beamline I24 of the Diamond Light Source (Harwell Science and Innovation Campus, Didcot, UK) at 100 K using a PILATUS3 6 M detector. The data sets were processed with XDS using xia2 as part of the CCP4 version 7.0 program suite (54–56). The phase problem was solved by molecular replacement using Phaser-MR, taking Protein Data Bank structure 5DE0, DyP type B from *V. cholerae* (10) or the WT *Kp*DyP structure described here. The models were further improved by iterative cycles of manual model building using Coot (57) and maximum likelihood refinement using PHENIX-Refine (58). PHENIX-Refine converts intensities into amplitudes using the French and Wilson algorithm (59). Final stages of refinement included translation libration screw parameters (for R232A) or anisotropic refinement of all atoms (for WT *Kp*DyP and D143A/R232A), isotropic *B*-factor model, the automated addition of hydrogens and water molecules, optimization of X-ray/ADP weight, and optimization of X-ray/stereochemistry weight. The model was validated with MolProbity (60, 61), and the final resolution cutoff was determined using paired refinement with PDB-REDO (62). Metal coordination was validated using the CheckMyMetal web server (www.csgid.org/csgid/metal_sites) ^3^ (63, 64). Figures were prepared with PyMOL. Atomic coordinates have been deposited in the Protein Data Bank under accession codes 6FKS, 6FL2, 6FKT, and 6FIY.

Dimensions of putative access channels were calculated using the PyMOL plugin CAVER version 3.0.1. To calculate the access channel, the heme iron position was used as starting coordinates, and heme was excluded. Minimum probe radius was 0.9, shell depth was set to 10, and shell radius was set to 9.

## Author contributions

V. P., K. N., M. B., H. M., G. M., and S. H. data curation; V. P., K. N., M. B., G. B., S. V. D., P. G. F., and S. H. formal analysis; V. P. validation; V. P. investigation; V. P. visualization; V. P., K. N., S. V. D., and C. O. writing-original draft; V. P., G. B., S. V. D., P. G. F., S. H., and C. O. writing-review and editing; G. B., K. D.-C., S. V. D., and C. O. supervision; S. V. D., P. G. F., and C. O. conceptualization; C. O. funding acquisition.

## Supplementary Material

Supporting Information

## Footnotes

3 Please note that the JBC is not responsible for the long-term archiving and maintenance of this site or any other third party hosted site.

## Footnotes

2 The abbreviations used are:

DyP: dye-decolorizing peroxidase
ABTS: 2,2-azinobis(3-ethylbenzothiazoline-6-sulfonic acid)
SEC: size-exclusion chromatography
HS: high-spin
LS: low-spin
ECD: electronic CD
OTTLE: optical transparent thin-layer spectroelectrochemical
DSC: differential scanning calorimetry
CW: continuous-wave
TMB: 3,3′,5,5′-tetrametylbenzidine
W: water
mT: millitesla.
