# Supplementary material for: Roles of distal aspartate and arginine of B-class dye-decolorizing peroxidase in heterolytic hydrogen peroxide cleavage
Source: J Biol Chem. 2018 Aug 2;293(38):14823–38. doi: 10.1074/jbc.RA118.004773 (PMC6153280; doi:10.1074/jbc.RA118.004773)
Supplement: Supporting Information [file supp_293_38_14823__index.html]

Roles of distal aspartate and arginine of B-class dye-decolorizing peroxidase in heterolytic hydrogen peroxide cleavage — Mechanism of Compound I formation in KpDyP — Roles of distal aspartate and arginine of B-class dye-decolorizing peroxidase in heterolytic hydrogen peroxide cleavage — Mechanism of Compound I formation in KpDyP — Supporting Information 

# Roles of distal aspartate and arginine of B-class dye-decolorizing peroxidase in heterolytic hydrogen peroxide cleavage

## Supporting Information

- Supporting Information - S1, alkaline transition of D143A; S2, CW-EPR spectra of Compound I; S3, reaction of Compound I with serotonin, TMB and ascorbate; S4, access channels of wt and variants; S5, stereo views of active site of wt and variants
