# Supplementary material for: Roles of distal aspartate and arginine of B-class dye-decolorizing peroxidase in heterolytic hydrogen peroxide cleavage
Source: J Biol Chem. 2018 Aug 2;293(38):14823–38. doi: 10.1074/jbc.RA118.004773 (PMC6153280; doi:10.1074/jbc.RA118.004773)
Supplement: Supporting Information [file supp_RA118.004773_139280_1_supp_173712_pcgrl4.pdf]

Supporting Information

Roles of distal aspartate and arginine of B-class dye-decolorizing peroxidase in heterolytic hydrogen peroxide cleavage

Vera Pfanzagl[1] , Kevin Nys[2], Marzia Bellei[3], Hanna Michlits[1], Georg Mlynek[4], Gianantonio Battistuzzi[5], Kristina Djinovic-Carugo[4], Sabine Van Doorslaer[2], Paul G. Furtmüller[1], Stefan Hofbauer[1], and Christian Obinger[1*]

From the [1]Department of Chemistry, Division of Biochemistry, BOKU - University of Natural Resources and Life Sciences, 1190 Vienna, Austria; [2]Department of Physics, University of Antwerp, Antwerp, Belgium; [3]Department of Life Sciences, University of Modena and Reggio Emilia, via Campi 103, 41125 Modena, Italy; [4]Department for Structural and Computational Biology, Max F. Perutz Laboratories, University of Vienna, 1030 Vienna, Austria; [5]Department of Chemistry and Geology, University of Modena and Reggio Emilia, 41125 Modena, Italy

Running title: *Mechanism of Compound I formation in KpDyP*

*To whom correspondence should be addressed: Christian Obinger: Department of Chemistry, Division of Biochemistry, BOKU - University of Natural Resources and Life Sciences, 1190 Vienna, Austria; christian.obinger@boku.ac.at; Tel. +43-1-47654-77273; Fax. +43-1-47654-77259.

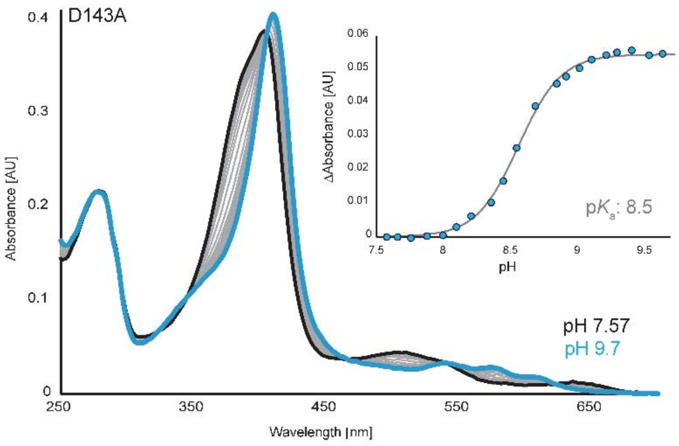

Figure S1. Alkaline transition of the D143A variant. The transition from high-spin ferric heme to hydroxo-ligated ferric heme of D143A was recorded between 250 and 700 nm at 25 °C. The mutant was prepared in 1 mL 50 mM phosphate buffer, pH 7.6, at a concentration of 5 µM. The pH was increased by adding small amounts of 1 M KOH. The pH was determined by adding equal amounts to a reference sample and measuring the pH using a WTW Inolab pH 720 pH electrode. The pK_a was determined from the inversion point of a sigmoidal fit of the differential absorbance at 410 nm using Sigma Plot [equation: $f = a/(1+\exp(-(x-x_0)/b))$].

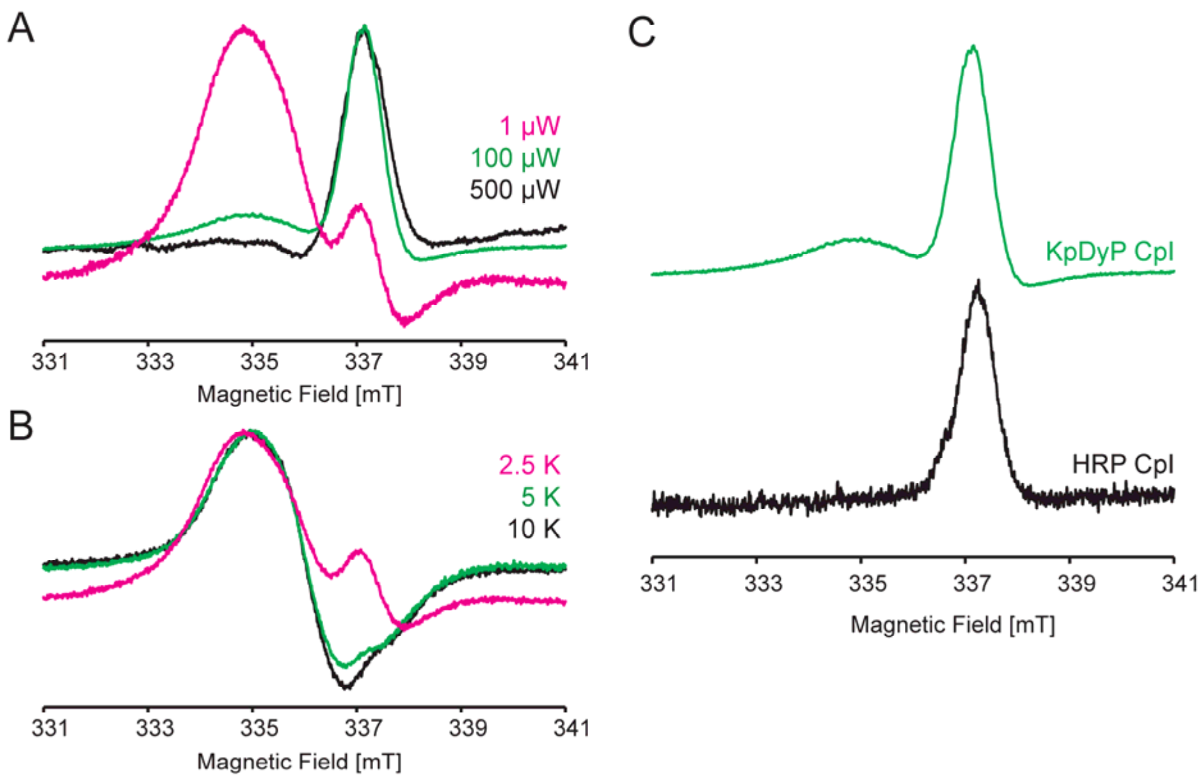

Figure S2. CW EPR radical signatures of Compound I state of wild-type *Kp*DyP and HRP. (A) Temperature dependence of X-band CW-EPR spectra (radical-type signal area) of the Compound I state of wild-type *Kp*DyP at 2.5 K. Microwave power was 1 μW (magenta), 100 μW (green) and 500 μW (black), (B) X-band CW-EPR spectrum (radical-type signal area) of the Compound I state of wild-type *Kp*DyP at a temperature of 2.5 K (magenta), 5 K (green) and 10 K (black), all recorded using a microwave power of 1 μW, (C) X-band CW EPR (radical-type signal area) of wild-type *Kp*DyP Compound I (green) and HRP Compound I (black) at 2.5 K. Microwave power was 200 μW for HRP and 100 μW for *Kp*DyP. Compound I was formed in all cases by addition of a 5-fold stoichiometric excess of hydrogen peroxide.

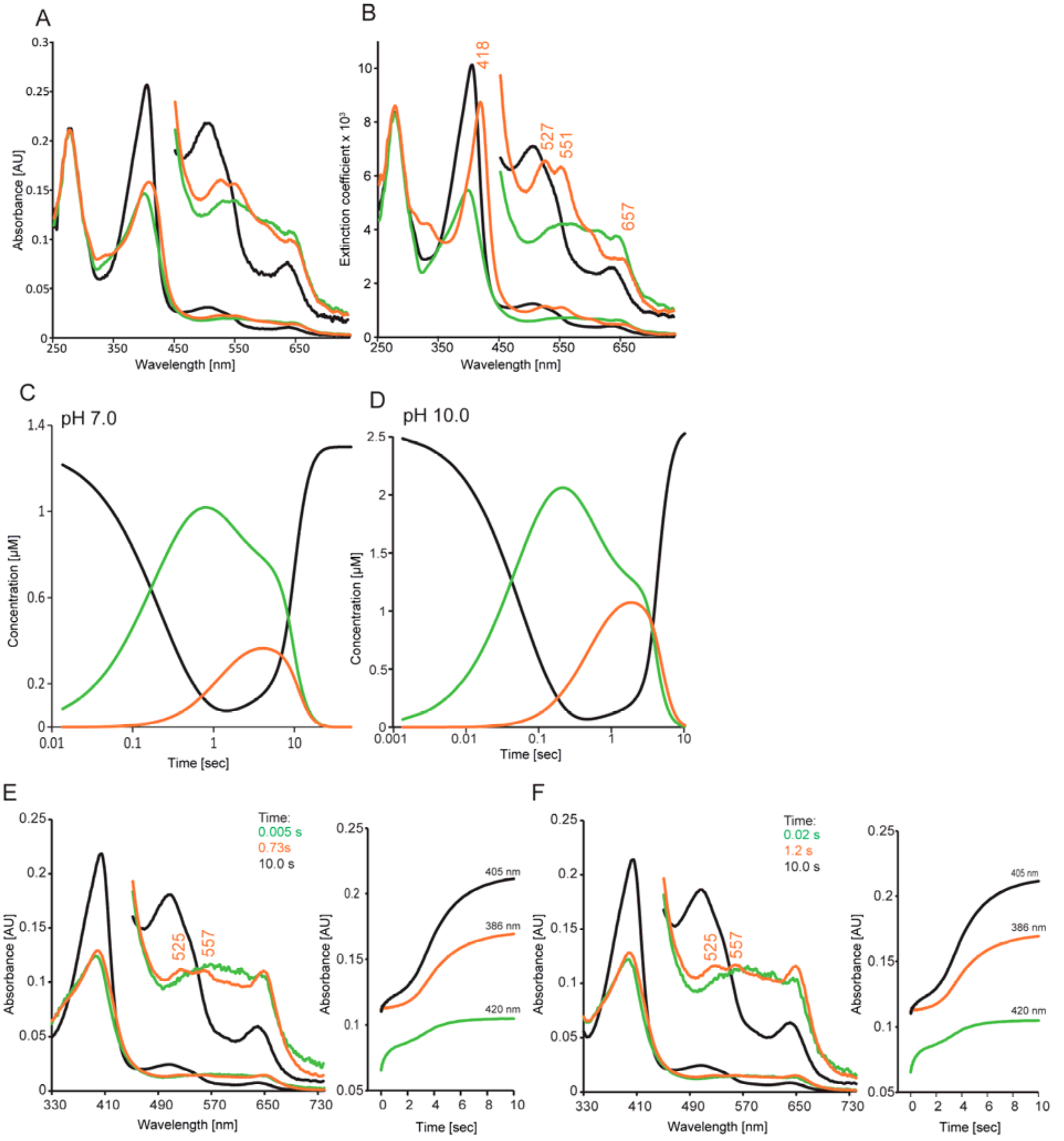

Figure S3. Reaction of wild-type *Kp*DyP Compound I with serotonin, TMB and ascorbate at pH 7.0 and pH 10.0. Ferric spectra are shown in black, Compound I spectra in green and oxoiron(IV)-type Compound II spectra are shown in orange. (A) Reaction of wild-type *Kp*DyP with hydrogen peroxide and serotonin (C) at pH 10.0 followed by the conventional stopped-flow mode. 4 μM enzyme (in 5 mM phosphate buffer, pH 7.0) was mixed with a two-fold

stoichiometric excess of hydrogen peroxide and 50 µM serotonin in 100 mM borate buffer, pH 10.0. (B) Simulated intermediate spectra using ProK and relevant absorption maxima. (C, D) simulated concentration profiles of the reaction of wild-type ferric *Kp*DyP with serotonin and hydrogen peroxide at pH 7.0 (C) and pH 10.0 (D), calculated using ProK. The ferric enzyme is shown in black, the Compound I intermediate in green and the oxoiron(IV)-type Compound II in orange. (E, F) Reaction of wild-type Compound I with tetramethylbenzidin (TMB) (E) and ascorbate (F) at pH 7.0 followed by the sequential stopped-flow mode. Compound I (green spectrum) was preformed by mixing 6 µM ferric *Kp*Dyp with 12 µM H_2O_2 in 50 mM phosphate buffer, pH 7.0. After 100 ms delay time 20 µM TMB and 200 µM ascorbate (E) or 2 mM ascorbate (F) were added. Relevant absorption maxima and time points of selection of the spectra are depicted.

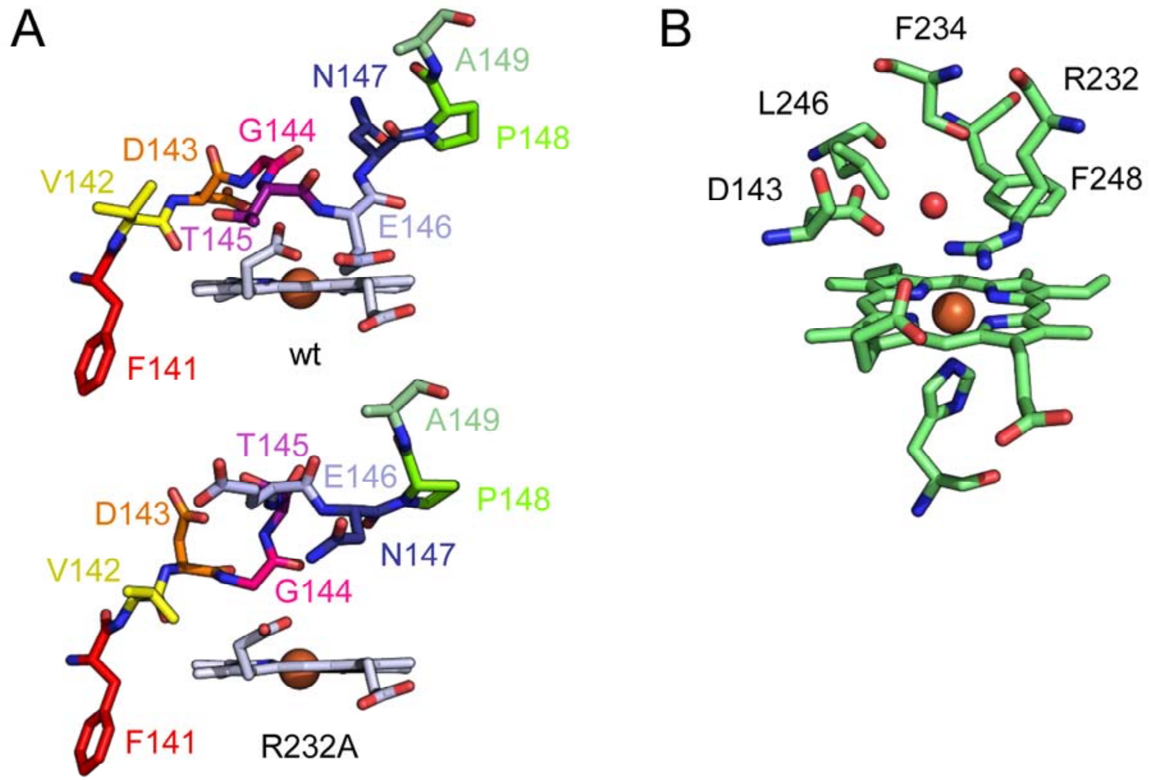

Figure S4. Access channel of wild-type *Kp*DyP and comparison of wild-type *Kp*DyP and R232A residues 141-149. (A) Comparison of residues 141 to 149 of wild-type *Kp*DyP (top) and the R232A variant (bottom). All residues and the heme *b* cofactor are shown as sticks, the heme iron is shown as an orange sphere. All residues are labeled in the corresponding color. (B) Residues forming access channel 1 in wild-type *Kp*DyP, all residues and the heme *b* cofactor are shown as green sticks, the distal water molecule W1 and the heme iron are shown as a red and orange sphere.

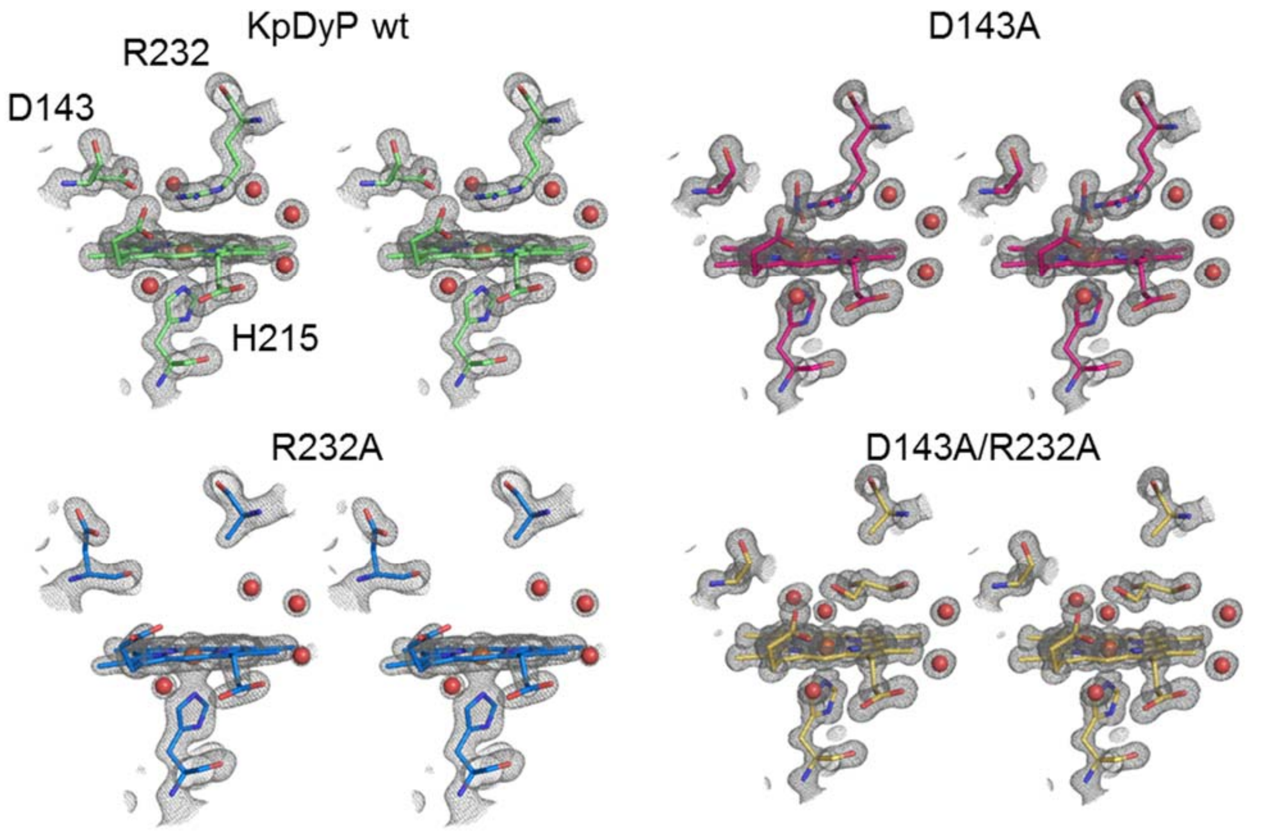

Figure S5. Stereo view of the active site of comparison of wild-type *Kp*DyP and variants with the $2F_o$-F_c electron density maps contoured at $\sigma = 1.5$. All residues, the heme *b* cofactor and relevant distal small molecules (nitrite and glycerol) are shown as sticks. The relevant water molecules and the heme iron are shown as a red and orange sphere. The $2F_o$-F_c electron density maps were contoured at $\sigma = 1.5$, within 1.6 Å of the selected atoms using pymol.
